# Transition metal coordination polymer-derived materials for supercapacitor applications: recent advances and future perspectives

**DOI:** 10.1098/rsos.250919

**Published:** 2025-08-13

**Authors:** Saifullahi Kabiru Sa’adu, Cheng Seong Khe, Muhammad Fadhlullah Abd. Shukur, Kwok Feng Chong, Chin Wei Lai, Kok Yeow You, Nik Roselina Nik Roseley, Eslam Aboelazm

**Affiliations:** ^1^ Department of Applied Science, Universiti Teknologi PETRONAS, Seri Iskandar, Perak 32610, Malaysia; ^2^ Center of Innovative Nanostructure and Nanodevices (COINN), Universiti Teknologi PETRONAS, Seri Iskandar, Perak 32610, Malaysia; ^3^ Universiti Malaysia Pahang Al-Sultan Abdullah, Gambang, Pahang 26300, Malaysia; ^4^ Universiti Malaya, Federal Territory of Kuala Lumpur 50603, Malaysia; ^5^ Universiti Teknologi Malaysia, Skudai, Johor 81310, Malaysia; ^6^ Universiti Teknologi MARA, Shah Alam, Selangor 40450, Malaysia

**Keywords:** transition metal coordination polymer-derived materials, supercapacitors, energy storage, electrode materials, electrochemical performance, synthesis methods

## Abstract

With the rising demand for efficient, sustainable and scalable energy storage, researchers are continuously exploring innovative materials for next-generation supercapacitors. Among these, transition metal coordination polymer (TMCP)-derived materials have emerged as promising candidates due to their high porosity, redox activity and structural adaptability. These materials offer significant potential for energy storage, but challenges like low electrical conductivity, structural instability and limited charge retention have restricted their widespread application. To overcome these hurdles, researchers have developed transformation strategies such as carbonization, phosphorization, sulfidation and oxide formation, enhancing the conductivity, stability and overall electrochemical performance of TMCPs. This review investigates the latest breakthroughs in TMCP-derived electrode materials, highlighting key advancements in synthesis techniques, structural engineering and hybrid material integration to improve charge transport and long-term durability. The incorporation of green chemistry principles, such as low-temperature synthesis, the use of non-toxic precursors, and strategies for recycling or reducing harmful byproducts, is highlighted, consequently promoting the fabrication of environmentally friendly supercapacitors. Furthermore, it explores how nanostructured designs and composite materials are unlocking new possibilities for high-performance supercapacitors. It also provides a perspective on the future of TMCPs in energy storage by fusing theoretical insights with experimental evidence. Theoretical frameworks like density functional theory and emerging machine learning models are other methods employed to better understand redox behaviour and drive material design. It addresses present issues and suggests viable future directions for their useful application.

## 1. Introduction

**Figure 1.**
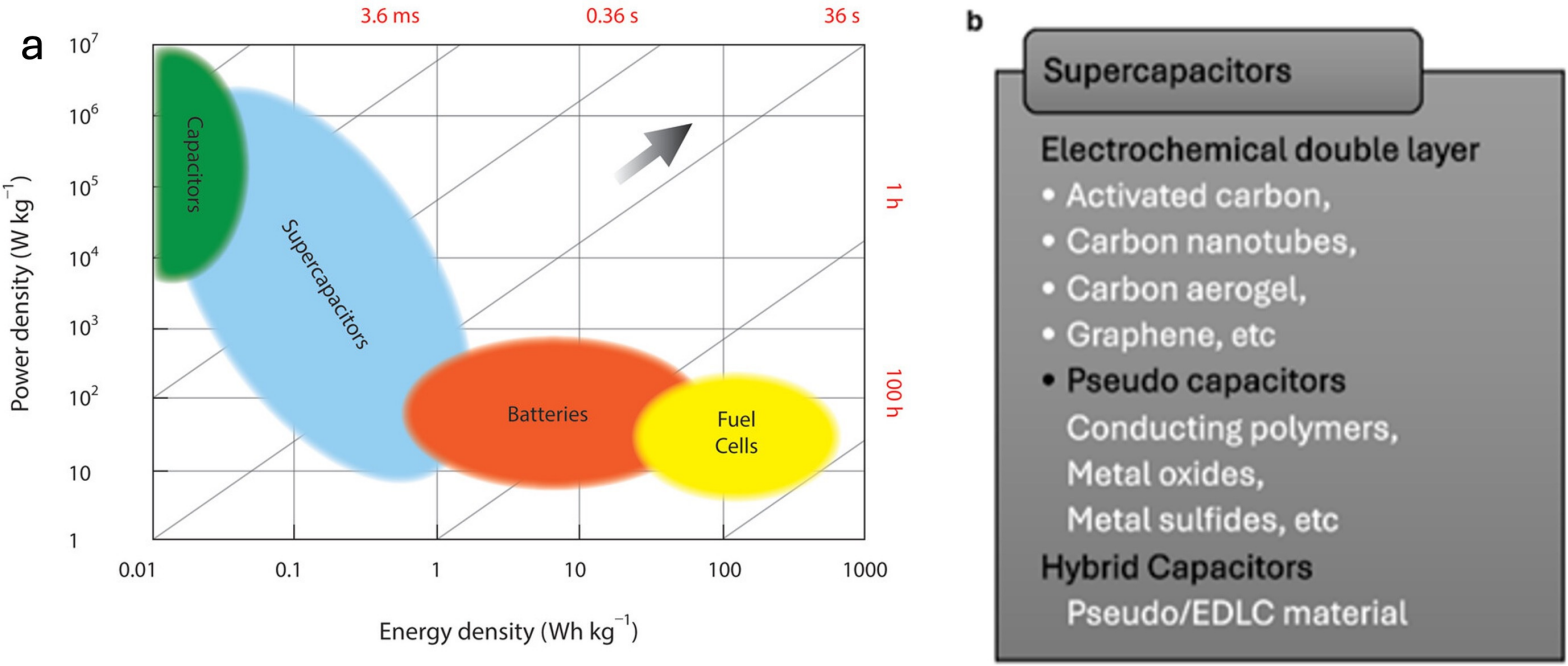
(a) A Ragone plot for the variation in power capabilities of different storage devices. Reproduced with permission. © 2022 John Wiley and Sons [6]. (b) Types of supercapacitors. Reproduced with permission. © 2020 Elsevier [7].

Conventional energy storage systems, such as batteries and capacitors, have been extensively utilized, each offering unique benefits and inherent limitations. Batteries are constrained by their low power output and short cycle life, despite their excellent energy density. Conversely, capacitors provide rapid power delivery but suffer from insufficient storage capacity. The performance disparity has driven the invention of supercapacitors, which are electrochemical devices that successfully bridge the performance gap between conventional batteries and capacitors. Supercapacitors, also known as electrochemical capacitors or ultracapacitors, have received a lot of interest due to their rapid charge–discharge rates, extended cycle life, high power and energy densities, great reliability and environmental sustainability, making them potential candidates for next-generation energy storage applications [1–5]. Figure 1a illustrates a Ragone plot, which compares the power and energy densities of various storage systems. The plot highlights how supercapacitors fall somewhere between high-power capacitors and high-energy batteries. The power densities of capacitors can reach up to 10⁶ W h kg⁻¹; however, batteries offer much higher energy densities. By striking a special balance between the two, supercapacitors increase their usefulness in a range of energy storage applications [4,6,8]. As one of the most promising electrochemical storage technologies, supercapacitors are known for their clean, renewable and sustainable operation, making them perfect for use in portable devices such as laptops, tablets and smartphones [2,4,5].

There are three main types of supercapacitors, as depicted in figure 1b, according to energy storage mechanism: (i) electrochemical double layer capacitors (EDLCs), (ii) pseudo capacitors (PCs) and (iii) hybrid capacitors (HCs) [5,7]. EDLCs generally use carbon-based electrode materials, such as activated carbon, nano-architected carbon, carbon aerogels and graphene. They store charge electrostatically; thus, no faradaic reaction occurs during the charge/discharge process in EDLCs. The structure of carbon-based materials, particularly their porosity and specific surface area, determines their double-layer capacitance. EDLCs have a low specific capacitance but good cycle life and power performance. These materials allow for the reversible adsorption/desorption of ions at the electrode/electrolyte interface, which leads to the accumulation of charge [1,2,4,5,9,10]. PCs, also known as redox capacitors, store charge using rapid and reversible faradaic processes enabled by transition metal oxides, hydroxides, sulfides and nitrides and conducting polymers. While these materials have a higher specific capacitance and energy density, they frequently have low cycling stability due to structural breakdown over multiple charge–discharge cycles [1,2,4,5,9–11]. Lastly, HCs combine EDLC and PC properties at either the material or device levels, increasing power and energy densities. They use complementary charge storage methods, leading to improved electrochemical efficiency. Nanoscale, porous electrode designs are regarded as a realistic solution to increase energy density and capacitance [1,2,4,5,9–11].

The performance of supercapacitors is majorly determined by the active electrode materials used. Although carbon-based materials, including graphene, carbon nanotubes and activated carbon, have been thoroughly investigated as supercapacitor electrode materials due to their large surface area, high stability and electrical conductivity [5,12], they primarily store charge through electric double-layer capacitance, which leads to a limited energy density and intrinsically low specific capacitance. These challenges reduce their application potential for high-energy storage systems in the future. Researchers are increasingly concentrating on materials with faradaic charge storage mechanisms, especially those that provide increased redox activity, like transition metal-based coordination polymers (TMCPs), in order to overcome these obstacles [5,11]. TMCPs present an appealing alternative by combining the redox-active properties of transition metals with the structural flexibility of coordination frameworks. Transition metals are particularly interesting in electrochemical applications due to their partially filled d-orbitals, which allow for several oxidation states and enable fast, reversible electron transfer reactions. These qualities make them ideal for use in supercapacitors, batteries and other energy storage devices [13–15].

**Figure 2.**
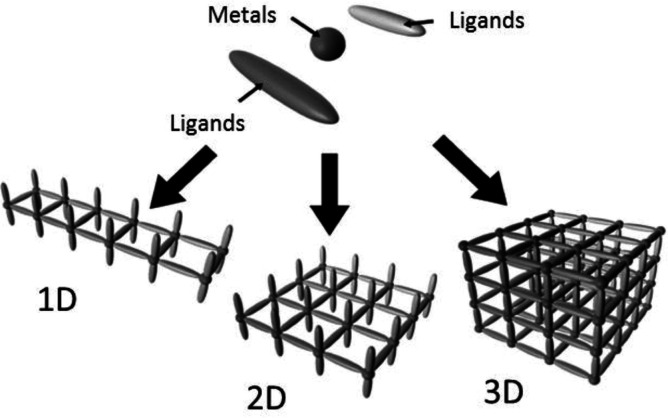
Coordination polymers depicted in one-, two- and three-dimensional structures. Reproduced with permission. © 2017 Elsevier [16].

Coordination polymers (CPs) are crystalline structures composed of metal entities (ions or clusters) and organic ligands linked through coordination bonds and can be used to create nanostructured functional inorganic materials with retained morphology and homogeneous size distribution as active electrode materials [1,14]. Their electrochemical performance is directly impacted by their dimensional tunability, which includes 1D nanowires, 2D nanosheets and 3D porous frameworks (figure 2). For example, the linear architectures of 1D CPs such as Co[Fe(CN)₆] nanowires possess excellent flexibility, which improve directional charge transmission. The layered architectures of 2D CPs such as Ni-HITP nanosheets offer large surface areas and stable mechanical properties, which enhance charge transfer and ion accessibility. While 3D CPs such as Fe[Fe(CN)₆] nanocubes form interconnected porous networks that offer stability and effective electrolyte penetration. These morphologies can be modified for gas storage, catalysis and supercapacitors by regulating the synthesis conditions [16,17]. These CPs can be transformed into highly conductive and electroactive materials via thermal or chemical modification (e.g. carbonization, phosphorization and sulfidation), overcoming the conductivity and stability problems associated with pristine CPs. TMCP-derived materials thus represent a diverse and promising family of supercapacitor electrodes, with variable chemistry, structural flexibility and improved pseudocapacitive behaviour [18,19].

Investigations into TMCP-derived materials for supercapacitors have increased dramatically in recent years, owing to the urgent need for high-performance energy storage systems that combine high energy and power densities [11]. Despite their promising redox activity and adjustable architectures, TMCPs often suffer from inherent constraints such as low electrical conductivity and relatively low cycling stability, necessitating structural and compositional changes to realize their full electrochemical potential [13,20]. Recent research shows that rational design of TMCP-derived materials, particularly through tactics like conductive carbon hybridization and heteroatom doping, can significantly improve their performance [21]. A Ni/Co-MOF-reduced graphene oxide (rGO) nanocomposite was reported to possess a specific capacitance of 860  F g⁻¹ at 1.0 A g⁻¹. An asymmetric AC/Ni/Co-MOF-rGO device delivered a specific energy of 72.8 Wh kg⁻¹ at 850 W kg⁻¹, maintaining 15.1 Wh kg⁻¹ at a high power of 42.5 kW kg⁻¹ with 91% efficiency. This study shows how carbonaceous frameworks improve conductivity and structural stability [22]. Similarly, heteroatom doping has been extremely beneficial. N, P co-doped activated carbon produced from TMCP precursors displayed a specific capacitance of 375 F g⁻¹ at 0.5 A g⁻¹ and retained 315.1 F g⁻¹ at 10 A g⁻¹. Cycling stability was exceptional, with 98.7% after 10 000 charge–discharge cycles. These case studies demonstrate how combining carbon integration and heteroatom functionality can result in materials with enhanced electron/ion routes, surface area and chemical activity [23].

**Figure 3.**
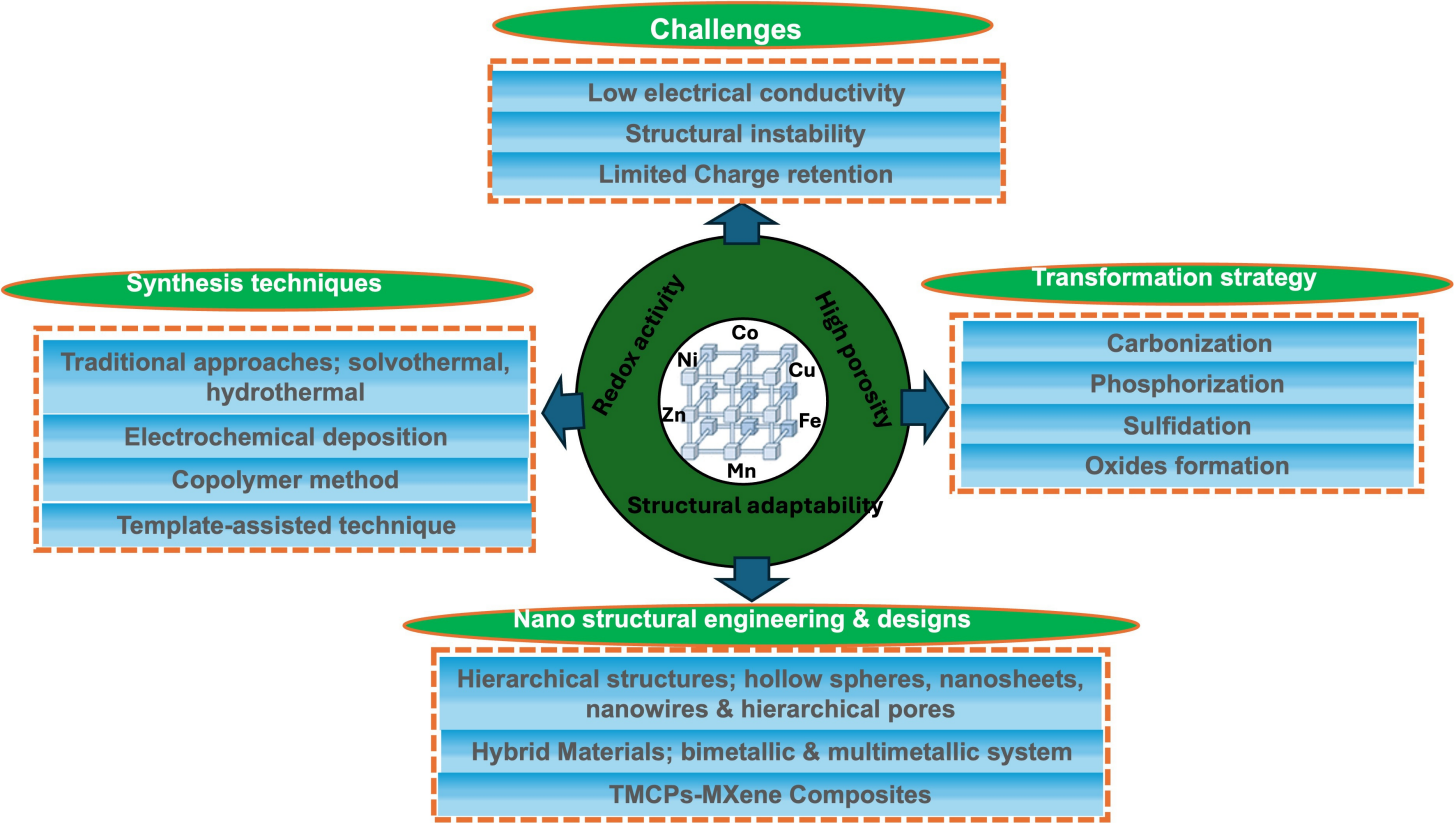
A schematic diagram of what this review article covers.

Furthermore, template-assisted synthesis and post-synthetic changes (e.g. sulfidation and phosphorization) have allowed for the development of hierarchically porous structures that improve electrolyte accessibility and ion diffusion [23,24]. These focused structural and compositional optimizations show that combining TMCPs with carbon frameworks and dopants is a potential technique for overcoming conventional performance limitations. This review article intends to provide a thorough and critical examination of recent advancements in the synthesis, modification and application of TMCP-derived electrode materials for supercapacitor applications (figure 3). Special emphasis is placed on studying design techniques, electrochemical performance evaluations, structural morphologies and future potential to steer the development of scalable, high-efficiency and sustainable energy storage solutions.

## 2. Overview of TMCPs as precursors for supercapacitors

### 2.1. Key benefits of TMCPs in energy storage applications

The growing usage of renewable energy sources and portable electronic gadgets drives the demand for effective energy storage systems. Supercapacitors are energy storage devices with faster charge/discharge rates, longer cycle lifetimes and higher power density than batteries. However, their poor energy density remains a significant impediment. CP-derived materials, particularly those produced from the thermal or chemical degradation of metal–organic frameworks (MOFs), are promising materials for supercapacitor electrode applications [11,19,25,26]. Transition metals like nickel (Ni), cobalt (Co), copper (Cu), zinc (Zn), iron (Fe) and manganese (Mn) are frequently incorporated into CPs due to their capacity to undergo reversible redox processes, which contribute to pseudocapacitance, and their compatibility with high-porosity architectures [11,20,27]. Table 1 summarizes the essential electrochemical characteristics of some transition metals (Ni, Co, Cu, Fe, Mn and Zn) frequently in use.

**Table 1.** Summary of the essential electrochemical characteristics of Ni, Co, Cu, Fe, Mn and Zn.

transition metals	redox activity	electrochemical performance	stability	ref.
nickel (Ni)	Ni²^+^/Ni³^+^ redox transitions contribute to high pseudocapacitance	moderate; prone to dissolution in alkaline electrolytes	high capacitance (approx. 2000 F g^−1^), good cycling stability	[27,28]
cobalt (Co)	Co²^+^/Co³^+^ transitions enable fast redox kinetics	high stability in alkaline media	high capacitance (approx. 1000–3500 F g^−1^), excellent rate capability	[27,29,30]
copper (Cu)	Cu²^+^/Cu^+^ redox pairs contribute to charge storage	poor stability due to dissolution in aqueous electrolytes	lower capacitance (approx. 500 F g^−1^) requires hybridization for stability	[27,30,31]
iron (Fe)	Fe²^+^/Fe³^+^ provides stable faradaic charge storage	high thermal and chemical stability	moderate capacitance (approx. 700–1000 F g^−1^), high reversibility	[27,32]
manganese (Mn)	Mn³^+^/Mn⁴^+^ oxidation states drive pseudocapacitance	moderate; prone to structural degradation	high specific capacitance (approx. 1500 F g^−1^), good cyclic stability with doping	[19,27,33]

**Figure 4.**
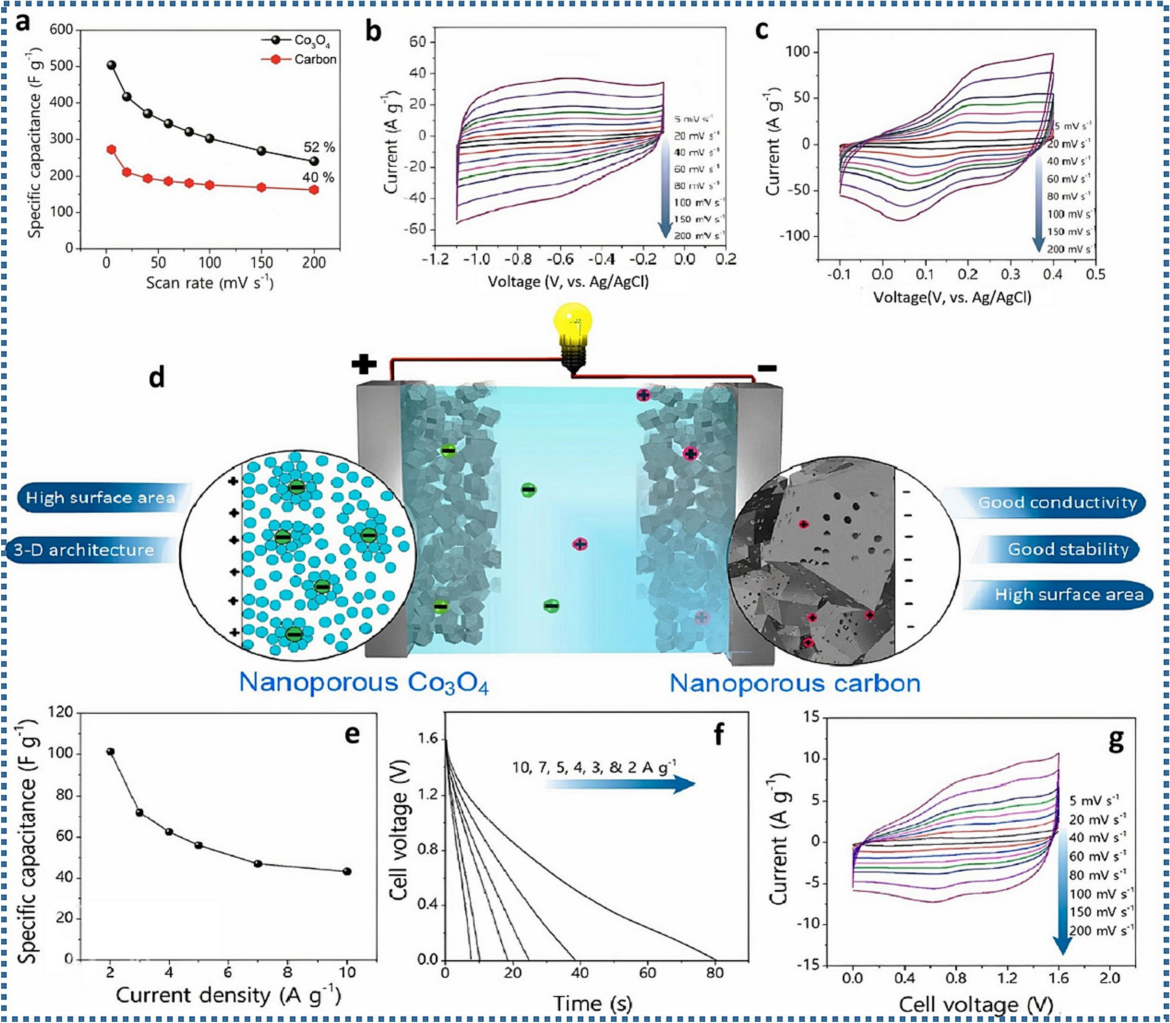
(a) The specific capacitance for carbon and nanoporous Co_3_O_4_ electrodes as a function of scan rate. CV profiles of (b) carbon and (c) Co_3_O_4_ nanoporous materials at different scan rates (5–200 mV s^−1^). (d) ASC cell schematic depiction using carbon as the negative electrode and nanoporous Co_3_O_4_ as the positive electrode. Specific capacitance dependence on applied current densities (e); GCD curves for the produced ASC cell at various current densities (f); and ASC CV curves (g). Reproduced with permission. © 2023 Elsevier [26].

Supercapacitors derived from TMCPs are energy storage devices that result from conversion of CPs to functional electrode materials. By utilizing the structural adaptability of CPs and the inherent benefits of transition metals, these materials improve performance parameters such as cycle life, energy density and power density of supercapacitors [34,35]. In addition, many TMCPs are synthesized using earth-rich and environmentally friendly precursors, supporting the principles of green chemistry [19,20]. Recent studies have focused on the development of CP-derived materials with optimized compositions, architectures and morphologies that yield high specific capacitance, improved cycling stability and scalability [26,36]. For example, Yang *et al.* synthesized hierarchical ZnO/NiO nanocomposites from Zn/Ni MOFs by adjusting the Zn/Ni molar ratio and calcination parameters. The study discovered that the hierarchical seaweed microflower ZnO/NiO performed better electrochemically when the Zn/Ni molar ratio was 1:1 and the temperature was 150°C achieved at a ramping rate of 50°C min^−1^. This structure improved the specific surface area (SSA) and ensured an optimal distribution of pore sizes. According to the electrochemical impedance spectroscopy (EIS) results, the prepared nanocomposite has low intrinsic and interfacial resistance due to the ZnO insertion. When utilized as a supercapacitor electrode, this nanocomposite has a specific capacitance of 435 F g^−1^ at 1.0 A g^−1^. After 900 cycles of 10 A g^−1^, retention is around 65% [26]. In another study by Zhang *et al.*, they developed zinc-ion hybrid micro-supercapacitors (MSCs) with activated carbon anodes and Zn nanosheet anodes. MSCs are miniaturized energy storage devices that combine the benefits of normal capacitors and batteries at the microscale. The produced hybrid device demonstrated a good areal energy density of 115.4 µWh cm^−2^ and power density of 0.16 mW cm^−2^. This enhanced performance is linked to the complementary storage mechanism: carbon-based cathodes allow quick adsorption of surface ions (capacitor types), while zinc anodes conduct reversible redox processes (battery types), which increase both energy and power output [36]. Salunkhe *et al.* produced Co_3_O_4_ polyhedra formed from ZIF-67 (figure 4). The resultant product has a polyhedral shape and a substantial SSA of around 148 m^2^ g^−1^. Interestingly, this SSA is more than that of Co_3_O_4_ generated using other standard processes such as chemical precipitation and the hydrothermal approach. To heat ZIF-67, the researchers used a twofold annealing method, resulting in porous Co_3_O_4_ polyhedra that preserved the original MOF’s distinctive structure and architecture. In the first procedure, these polyhedra were calcined at 500°C in a N_2_ environment, followed by annealing at 350°C at 5°C min^−1^. Calcination in N_2_ provided considerable SSA while preserving the polyhedral shape [26].

### 2.2. Various transition metals used in TMCP materials for supercapacitor applications

**Figure 5.**
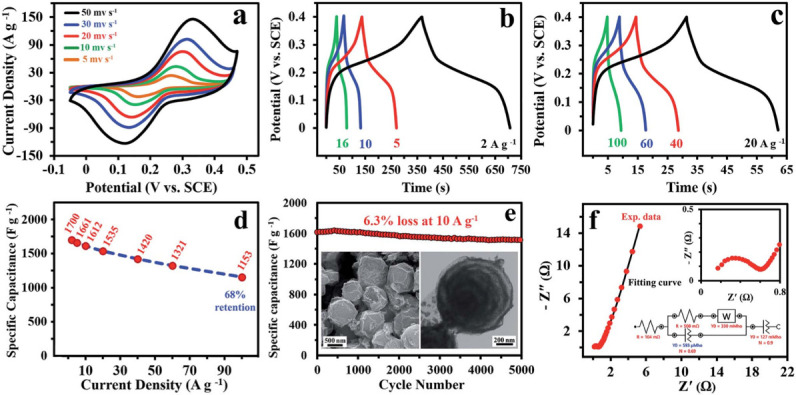
Evaluation of the electrochemical performance of the ON-CCO electrode in a three-electrode cell (a–f). Reproduced with permission. © 2018 Royal Society of Chemistry [52].

The highly configurable porosity of TMCPs, variable oxidation state potential and precision metal–ligand design provide versatility for energy storage customization. TMCPs have shown promising results for enhanced pseudocapacitive supercapacitors. Transition metals govern the redox activity, stability and electrochemical properties [8,11,20,27,36,37]. A wide range of Ni-based materials such as MOFs, perovskite, hydroxides, bimetallic compounds, oxides, sulfides, phosphides, phosphates, nitrides, carbides, borides, silicides and Ni metal were produced. These materials have outstanding pseudocapacitance due to high redox activity (Ni²^+^ ↔ Ni³^+^) and high specific capacitance above 1000 F g^−1^ [27,28]. Cobalt (Co)-based materials are distinct electrode materials frequently employed in energy storage systems. Co-based materials are mostly found as oxides, hydroxides or composites with other metals. They have superior pseudocapacitive qualities, and high theoretical capacitance (approx. 3460 F g^−1^). Numerous accessible oxidation states (Co²^+^/Co³^+^) give them electrochemical activity by facilitating effective charge transfer and reversible faradaic processes [27,29,30]. Ni and Co are among the most intensively investigated transition metals because of their high redox activity and ability to make stable bimetallic complexes. Synergistic effects in Ni–Co systems result from complementary electronic interactions: nickel provides stability and structural integrity, while cobalt delivers strong redox activity. This synergy improves overall conductivity and electrochemical reversibility by encouraging electron delocalization between the two metal centres, which in turn improves charge transfer kinetics [35,38,39]. After 20 000 cycles, Ni–Co bimetallic materials developed from coordination polymers with ‘onion-like’ hierarchical morphologies display specific capacitance values of up to 1900 F g⁻¹ and cycling retention above 93% [40,41]. Capacitances as high as 1377.5 F g⁻¹ have also been achieved by hollow or nanosheet Ni–Co oxides and sulfides, which often perform better than single-metal systems [19,42]. Trimetallic and multicomponent systems are gaining popularity, with researchers increasingly investigating combinations such as Ni–Fe–Mn. The performance of trimetallic systems, like Ni–Fe–Mn, is further enhanced by multi-electron redox processes, in which each metal contributes unique redox behaviour. Manganese offers structural variety and oxygen evolution kinetics, iron provides inexpensive redox activity and nickel improves cycle stability. Improved charge storage behaviour and electrochemical kinetics are favoured by the dispersion of electron density among metal centres. Spinel NiFe/Mn oxides made from TMCPs, for example, showed a capacitance of 1640 F g⁻¹ with 94% retention over 5000 cycles [43]. Similarly, the integration of ternary composites, such as Ni–Co phosphides, selenides and sulfides, with porous carbon-based frameworks further improves electrolyte interaction and increases electronic conductivity because of more delocalized electrons inside the crystal lattice [44–46]. Iron, the fourth most prevalent element on Earth, has various valence states (Fe⁰/Fe²^+^/Fe³^+^) that are ideal for pseudocapacitive reactions. Its extensive redox chemistry and sustainable nature make it suitable for long-term applications, despite having a lower capacitance than Co or Ni [27,32]. Manganese-based nanostructures, commonly found as oxides or metal composites, have a large surface area, structural flexibility, and high pseudocapacitive response due to the Mn³^+^/Mn⁴^+^ redox pair. However, carbon incorporation or heteroatom doping can greatly improve their naturally low conductivity [19,27,33]. Heteroatom doping provides electron-rich or electronegative sites to the carbon matrix or TMCP structure, particularly when nitrogen or sulfur are present. By reducing the bandgap and increasing carrier density, this transformation modifies the electrical structure, resulting in improved conductivity, active site exposure and electron mobility. For example, nitrogen-doped Mn-based CPs showed improved electrochemical interface and improved charge transfer kinetics, which resulted in increased specific capacitance [47,48]. Cu-based materials are appealing because they are affordable, environmentally stable and have strong conductivity. When combined with Ni or Co, Cu improves overall charge transfer and catalytic activity, although having a weaker pseudocapacitive behaviour on its own. Cu–Co oxides, particularly those originating from TMCPs, have an ultrahigh capacitance (approx. 1700 F g⁻¹) and form hollow, onion-like spheres with low internal resistance [27,30,31]. Despite their low redox activity, zinc-based products are affordable and safe for the environment. Their surface area and nanostructure have an impact on their electrochemical behaviour. In hybrid systems, zinc is frequently used as a stabilizing element to improve rate performance and structural durability. When zinc was added to Ni/Co-MOF-derived electrodes, one study demonstrated enhanced rate capability and electrochemical retention [27,49–51]. Copper and zinc systems have also been incorporated into CPDMs, typically in conjunction with Ni or Co. Figure 5 shows the onion-like nanoporous CuCo_2_O_4_ which is a promising electrode material for high-performance supercapacitors because of all these exceptional electrochemical properties including high reversibility, fast kinetics, low internal resistance and an ultrahigh specific capacitance. Cu–Co oxide materials produced from CP precursors formed onion-like hollow spheres with a specific capacitance of 1700 F g⁻¹ and exhibited competitive electrochemical behaviour [52]. The prospective for optimizing TMCP-derived materials by electronic engineering (e.g. doping) and compositional tuning (e.g. multi-metal systems) offers a viable route to fabricating outstanding performance, scalable supercapacitor electrodes [53].

## 3. Preparations of TMCP-derived materials for supercapacitors

### 3.1. Traditional methods for the preparation TMCPs

**Figure 6.**
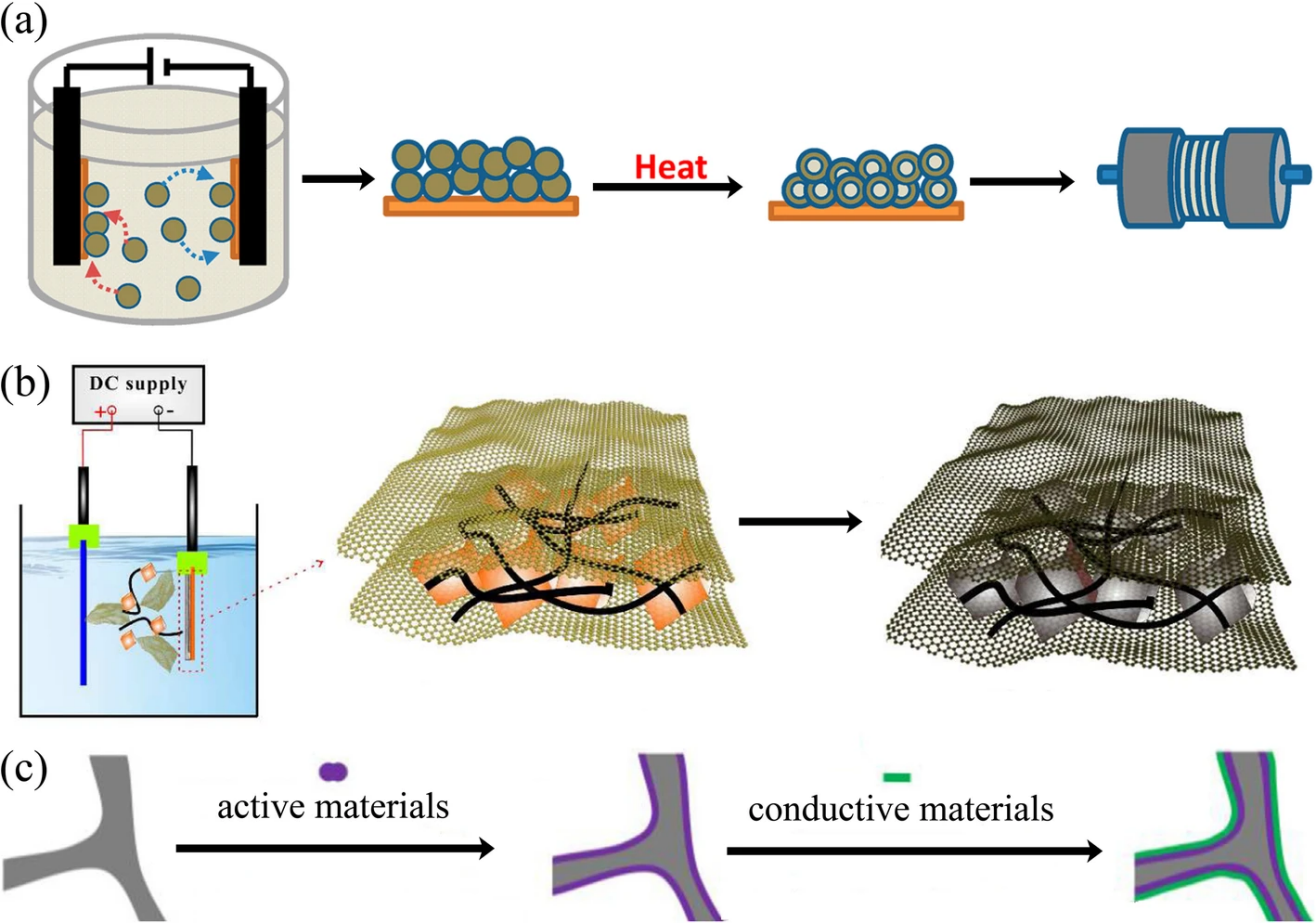
(a) Schematic diagram for fabrication of binder-free, carbon-free film electrodes. (b) Fe_3_O_4_/CNTs/rGO composite electrode production process. (c) The synthesis route for rGO/active materials/Ni foam. Reproduced with permission. © 2020 Springer Nature [58].

Traditional techniques such as solvothermal and hydrothermal are the most employed methods for the preparation of TMCPs. Precise synthesis of TMCPs and their derivatives through solution-based reactions allows control over crystal orientation, hierarchical morphologies and phase uniformity. These techniques frequently produce CPs with specific morphologies, including hierarchical frameworks, nanorods and nanosheets [54,55]. MOFs with redox active metal ions and a wide range of organic linkers have been extensively studied. A simple one-pot solvothermal technique was used to make hollow Ni/Co-MOFs, with the size of the projections on the MOF’s surface controlled by the molar ratio of metal ions and trimesic acid linkers. The small projections provide numerous redox sites for electrochemical reactions, and the hollow spheres should provide sufficient contact area for the electrolyte to reduce ion transport resistance. Ni/Co-MOFs function well in supercapacitors. The Ni/Co-MOF with a 1:1 ratio (metal ions:trimesic acid linkers) has exceptional performance, with a specific capacity of 828 C g^−1^ at 1 A g^−1^ and a rate capability of 68% at 20 A g^−1^ [56]. Morphology has a significant influence on ion diffusion, electron transport and structural stability in supercapacitor electrodes. Recent research has shown substantial success in building hierarchical and nanoscale morphologies from TMCP precursors. Hollow capsules, nanocages and core–shell designs increase ion-accessible surface areas, resulting in improved electrolyte penetration and ion transport [56]. The hydrothermal process was used to prepare spinel-type zinc/nickel-based electrode materials on nickel foam (ZnNi_2_O_4_/NF) with highly porous structures and well-organized active sites by adjusting the temperature and duration of the reaction, as well as an appropriate calcination step. At a current density of 1 A g^−1^, ZnNi_2_O_4_/NF has a significantly greater energy density (386 C g^−1^). After 1500 cycles at 10 A g^−1^, ZnNi_2_O_4_/NF retains 83% of its initial capacity. ZnNi_2_O_4_/NF has a high energy density of 32.15 Wh kg^−1^ and a power density of 317 W kg^−1^, outperforming earlier electrode materials based on Zn and Ni for supercapacitor applications [57]. Using electrochemical deposition, TMCPs can grow directly on conductive substrates, improving mechanical stability and lowering interfacial resistance. Cu-based CPs have been demonstrated to improve overall conductivity and durability by facilitating binder-free electrode manufacturing through direct electrodeposition onto conductive substrates as illustrated in figure 6 [58].

### 3.2. Advanced techniques for the preparation TMCPs

**Figure 7.**
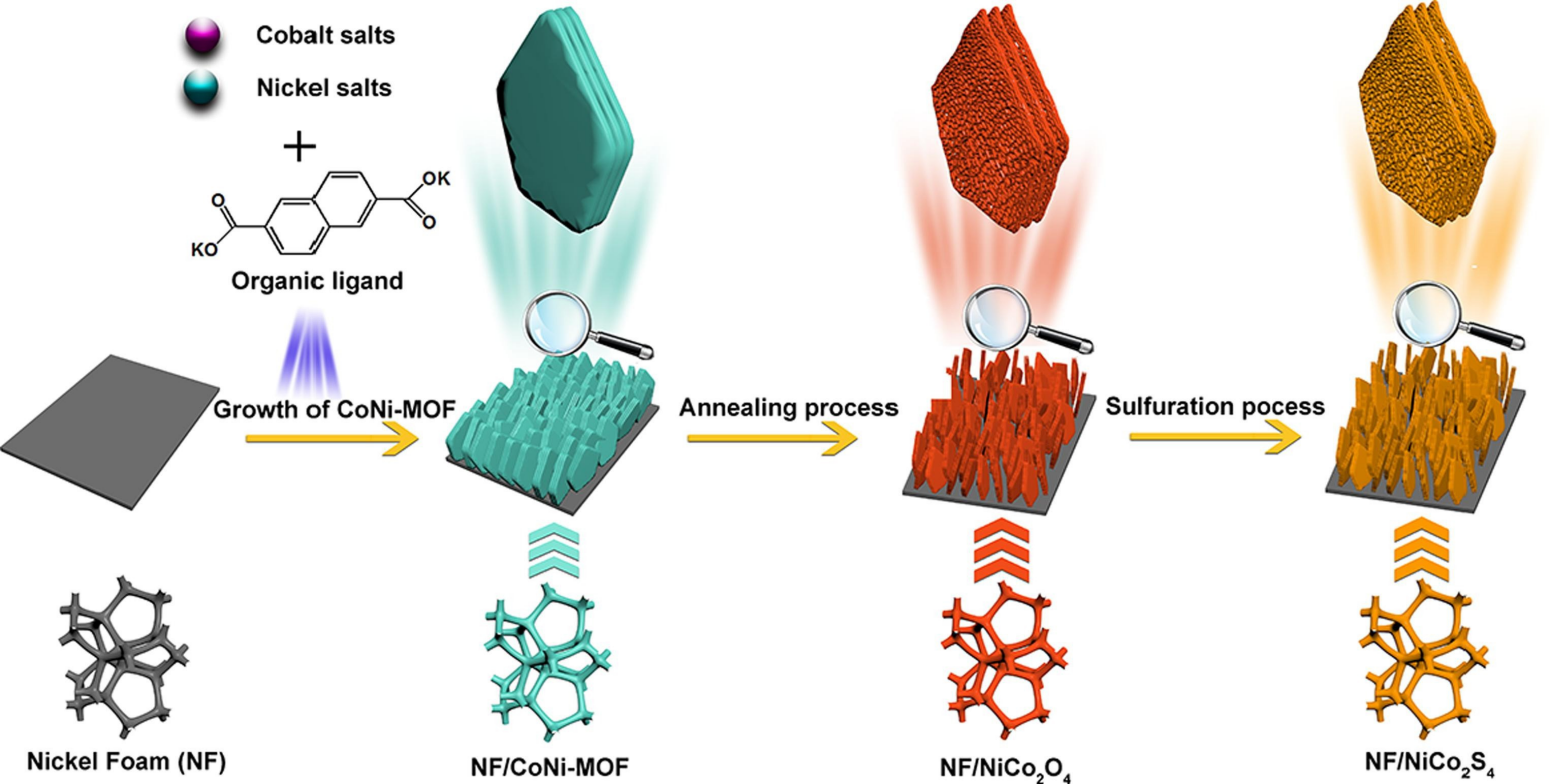
Schematic illustration of the formation of porous NiCo_2_S_4_ nanosheets from CoNi-MOF solid sheets. Reproduced with permission. © 2021 Elsevier [59].

Innovative synthetic approaches are being investigated more and more. Recent developments transform pristine MOFs into highly conductive derivatives by post-synthetic changes such as phosphorization and sulfurization. These developments allow for strong structural integrity and high redox potential by carefully adding phosphides or sulfides to materials generated from TMCP. The sulfidation of bimetallic MOFs shown in figure 7 enhanced the electronic conductivity, like Ni–Co, reaching >1300 F g⁻¹. Fast electrolyte penetration and abundant electrochemical active sites are provided by porous NiCo_2_S_4_ nanosheet arrays, which exhibit exceptional cycling stability with an 82.6% retention after 10 000 cycles [59].

**Figure 8.**
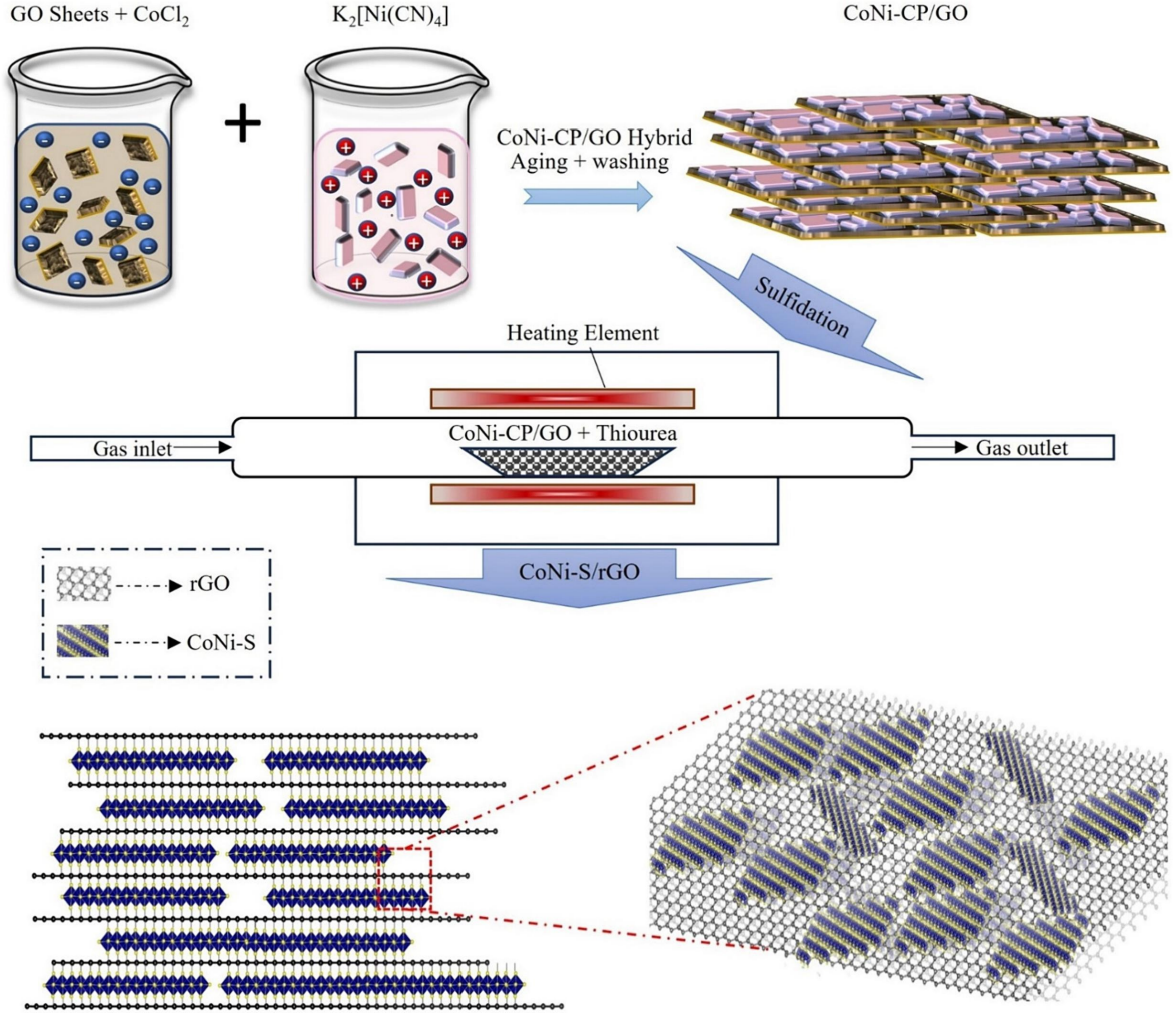
Schematic representation of the formation of CoNi-S/rGO. Reproduced with permission. © 2024 American Chemical Society [60].

To increase the conductivity and electroactive surface area for hybrid supercapacitors, a study by Aboelazm *et al.* presents a novel cathode material made of reduced graphene oxide (rGO) sheets and CoNi sulfide (CoNi-S) nanostructured flakes that are connected by adding sulfur atoms. The emergence of a layered structure of CoNi-S/rGO shown schematically in figure 8, in which rGO sheets enclose CoNi-S flakes, produced remarkable results. The CoNi-S/rGO composite demonstrated a specific capacitance of 3308 F g⁻¹ (1654 C g⁻¹) at 1 A g⁻¹ [60].

**Figure 9.**
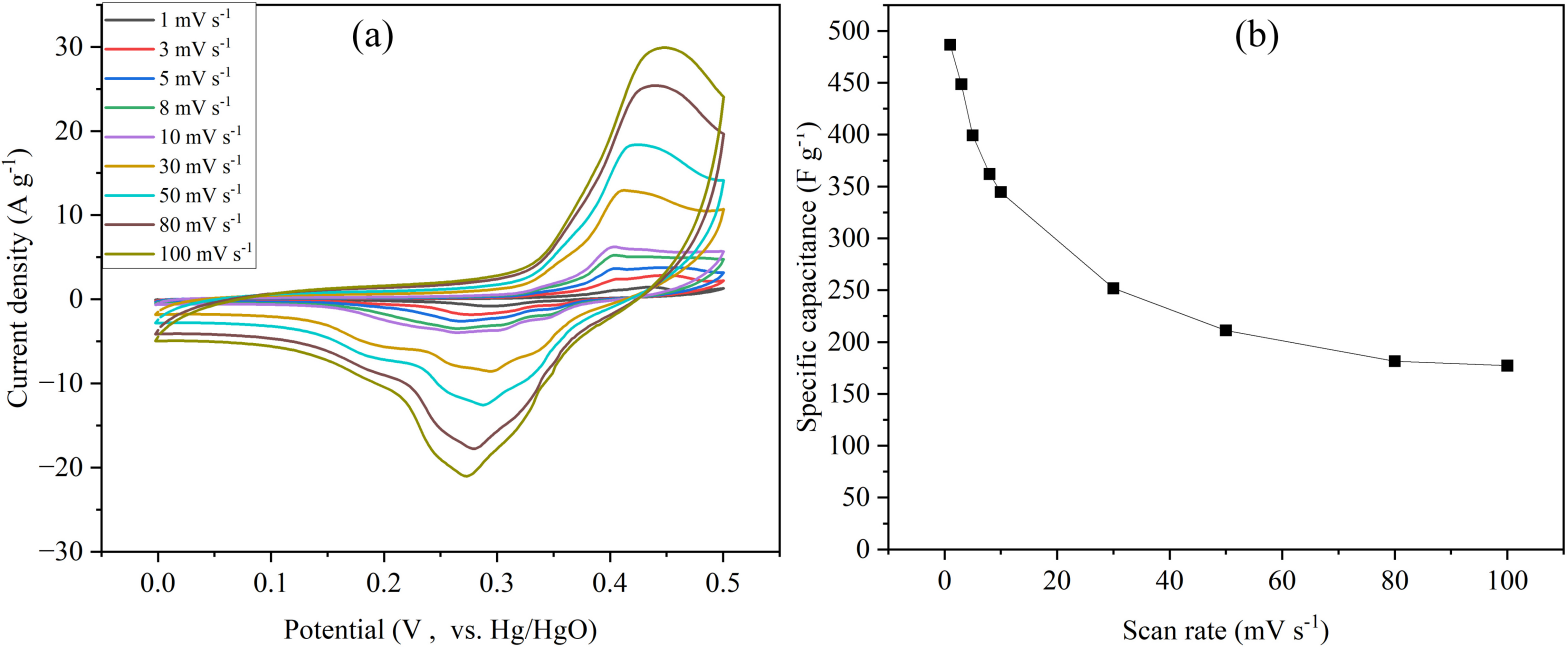
(a) Cyclic voltammetry at different scan rates, and (b) specific capacitance at different scan rates. Reproduced with permission. © 2024 Trans Tech Publications Ltd [1].

A functional heterostructure (NiCoP-MOF) comprising nickel/cobalt-MOF (NiCo-MOF) and phosphide (NiCoP) was devised and manufactured by localizing phosphorization of unique lamellar brick-stacked NiCo-MOF assemblies generated by a hydrothermal technique. The NiCoP-MOF-based hybrid supercapacitor has high capacity (226.3 C g⁻¹), energy density (50.3 Wh kg⁻¹) and durability (about 100% capacity retention over 10 000 cycles). This *in situ* heterogenization method highlights electrical structure modulation while preserving well-defined porosity and morphology, making it promising for building MOF-based derivatives for high-performance energy storage devices [61]. A study presents a novel approach to synthesizing efficient electrode materials using the copolymer method. The Co_3_C/rGO nanocomposite has a unique porous hollow cubic morphology, which was not previously tested for electrode materials synthesized using this method. This distinctive hollow cubic structure considerably increases the electrode’s porosity, allowing for better ion insertion and extraction during the charging and discharging operations. From figure 9, the synthesized Co_3_C/rGO electrode demonstrated remarkable electrochemical performance with a specific capacitance of 486.6 F g^−1^ at 1 mV s^−1^ and a low internal resistance of 0.58 Ω [1].

**Figure 10.**
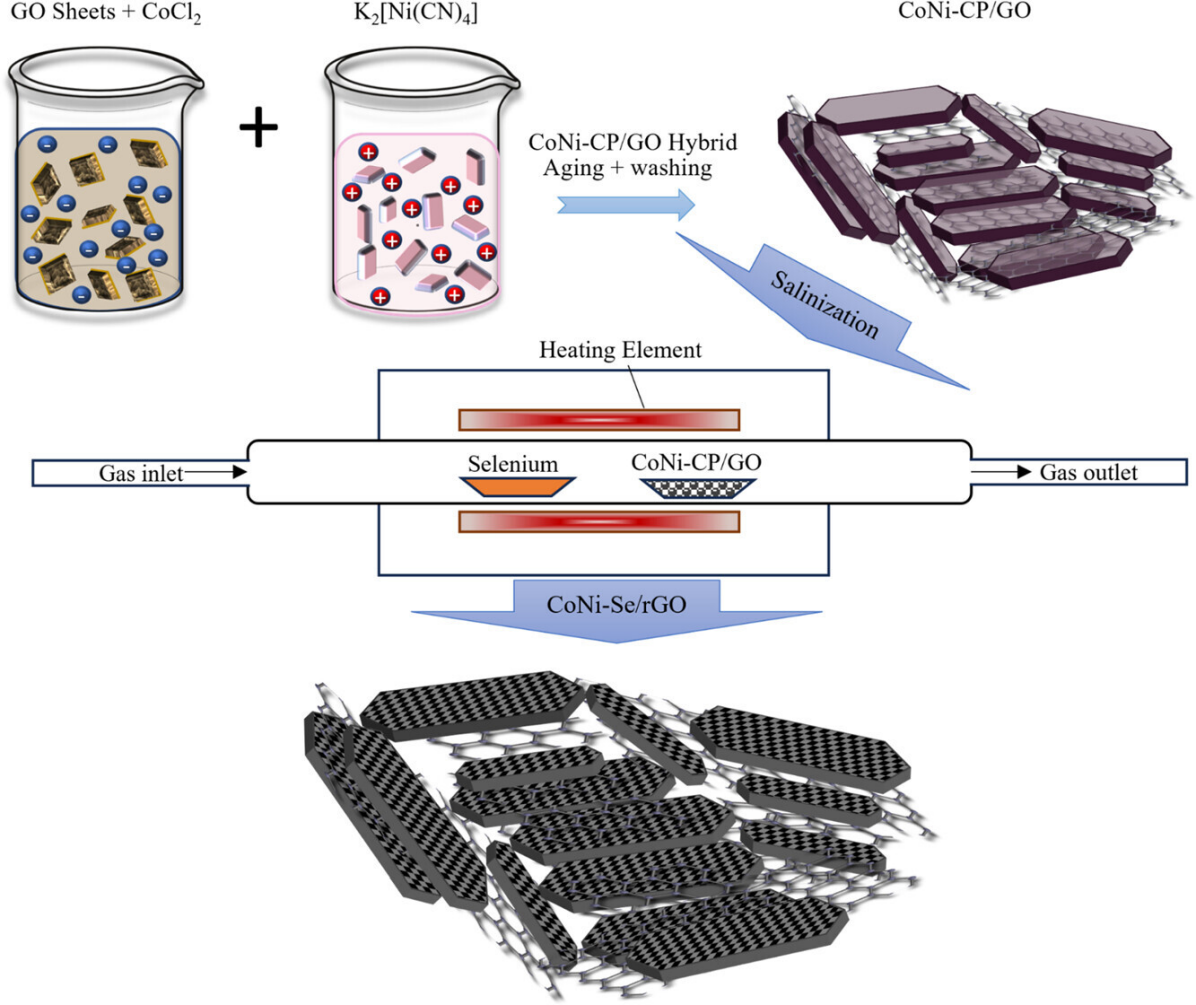
Schematic representation of the formation of CoNi-CP/GO precursors and their thermal conversion into CoNi-Se/rGO composite in the presence of Se powder. Reproduced with permission. © 2021 American Chemical Society [63].

The combination of metal cores and reduced graphene oxide features in a cold plasma atmosphere resulted in a hierarchical three-dimensional nanostructure of Ni/Co-MOF@rGO electrodes with more electroactive sites with a record-breaking 3093 F g^−^¹ capacitance, which was beneficial for high-efficiency redox processes. This study clearly describes the cold-plasma synthesis of MOF nanocomposite with the requisite morphological properties for energy storage applications [62]. Research by Aboelazm *et al.* investigates the potential of transition metal selenide (TMSe), recognized for its high activity, electrical conductivity and stability, in energy storage and conversion applications. It strategically harnessed the synergistic advantages gained from developing hollow structures of binary metal selenide (CoNi-Se) at the surface of reduced graphene oxide (rGO) arrayed in a 3D morphology (CoNi-Se/rGO). Figure 10 shows a schematic representation of the formation of CoNi-CP/GO precursors and their thermal conversion into CoNi-Se/rGO composite in the presence of Se powder. The CoNi-Se/rGO electrode exhibits significant faradic behaviour, resulting in a specific capacitance of 2957 F g^−1^ (1478.5 C g^−1^), surpassing the value of 2149 F g^−1^ (1074.5 C g^−1^) at a current density of 1 A g^−1^. Both materials preserve 83% of capacitance at 10 A g^−1^ compared with 1 A g^−1^. The two-electrode coin cell system has a high energy density of 73 Wh kg^−1^ and a power density of 1500 W kg^−1^, with 90.4% capacitance retention even after 20 000 cycles. This study highlights the potential of CoNi-Se/rGO composite as a high-performance electrode material for energy storage [63].

Widely used template-assisted procedures use a template that establishes the desired form and porosity to produce materials with precisely defined porous structures. With this technique, multilayer porosity may be achieved, allowing for the engineering of varying pore sizes and distributions to improve functioning in a range of applications. Multi-level pore architectures substantially shorten ion diffusion lengths and mitigate charge accumulation restrictions [64]. In this approach, the reactants self-assemble into polymers with the template agent serving as the configuration’s basic component. The template is removed from the resulting entity to produce a layered double hydroxide (LDH) material with accurate morphological and dimensional properties. It must consider the LDH’s shape, size, and functional properties. NiCo LDH was synthesized on metallic porous carbon (Ni@NC) with a distinctive cauliflower form using a twofold template approach. Ni-MOF carbonization was used to create the porous carbon, which was subsequently grafted with Co-ZIF. Finally, Co-ZIF is etched with nickel ion and the template is removed with hexamethylenetetramine to yield the final product, which has several active sites that contribute to the quick uptake and release of electrical charges in electrochemical processes [65].

Significant differences that affect material performance and application potential are shown by the comprehensive analysis of traditional and advanced TMCP preparation techniques (table 2). Because of their simplicity, scalability and affordability, traditional techniques like solvothermal, hydrothermal and electrodeposition procedures continue to be fundamental in the production of materials with regulated morphology. But when it comes to improving electrochemical characteristics, compositional diversity and fine structural adjustment, they frequently fall short. Advanced synthesis techniques, on the other hand, such as cold-plasma synthesis, copolymer methods, template-assisted techniques and post-synthetic modifications like phosphorization and sulfurization, provide more control over morphology, porosity and active site distribution, which enhances conductivity, redox activity and supercapacitor performance in general. Despite these benefits, advance technologies may involve hurdles in terms of process complexity, reproducibility, cost and scalability for industrial applications. As a result, future research should focus on integrating classic and new processes to create hybrid fabrication strategies that maximize performance while remaining feasible. Furthermore, many of these techniques do not address sustainability considerations such as energy usage, environmental effect or material reusability. A more quantitative comparison, including variables such as yield, energy density, specific capacitance and production cost, may provide useful information for selecting synthesis pathways customized to certain supercapacitor applications.

**Table 2.** Comparison of traditional and advanced TMCP preparation methods.

methods	type	description	advantages	disadvantages	ref.
solvothermal	traditional	uses organic solvents at high temperatures and pressures to create well-defined TMCP structures	— excellent crystallinity and homogeneous morphology — suitable for a wide range of MOFs — allows for variable particle size	— a lot of energy is consumed — prolonged response time — in certain circumstances, hazardous solvents are used	[55,66–68]
hydrothermal	traditional	uses water as the reaction medium at high temperatures and pressures, much as solvothermal	— eco-friendly due to water-based synthesis — provides control over size and form — improves the stability of TMCP structures	— special autoclaves are required — some precursors are only partially soluble in water — the process is time-consuming	[66,69]
electrodeposition	traditional	uses an electric current to cause TMCPs to settle on conductive surfaces	— direct deposition on electrodes increases adhesion — scalable and energy efficient — a rapid and regulated growing process	— there is limited control over structural complexity — further treatment may be required to improve characteristics	[58,70]
post-synthetic changes (phosphorization, sulfurization, selenization)	advanced	uses chemical vapour or solid-state reactions to convert TMCPs into metal phosphides or sulfides	— improves conductivity and electrochemical activity — increases stability and redox properties — ideal for energy storage applications	-multiple processing steps are necessary. Utilization of dangerous gases (PH_3_, H_2_, S). Controlling the final composition is challenging	[60,61,63,71,72]
copolymer method	advanced	polymeric frameworks are used to guide TMCP production, hence enhancing structural flexibility	— provides superior structural control — improves electrochemical characteristics by controlling polymer–metal interactions — offers improved mechanical stability	— selecting the right polymer is crucial — impurities may be introduced — synthesis techniques are complex	[1,73]
cold-plasma synthesis	advanced	plasma-assisted processes are used to alter TMCPs at low temperatures, hence increasing surface chemistry	— allows for fast synthesis at low temperatures — improves surface characteristics and reactivity — eco-friendly	— needs specialized equipment — there is limited scalability — expensive operating costs	[62,74,75]
template-assisted methods	advanced	sacrificial templates (such as silica and polymers) are used to generate hierarchical or porous TMCP structures	— increases surface area and porosity — improves electrolyte diffusion — enhances electrode performance	— more procedures are needed to remove the template — defects may be introduced — the complexity of synthesis is increased	[64,65,76]

## 4. Morphological engineering and performance optimization

### 4.1. Impact of morphology on electrochemical performance

**Figure 11.**
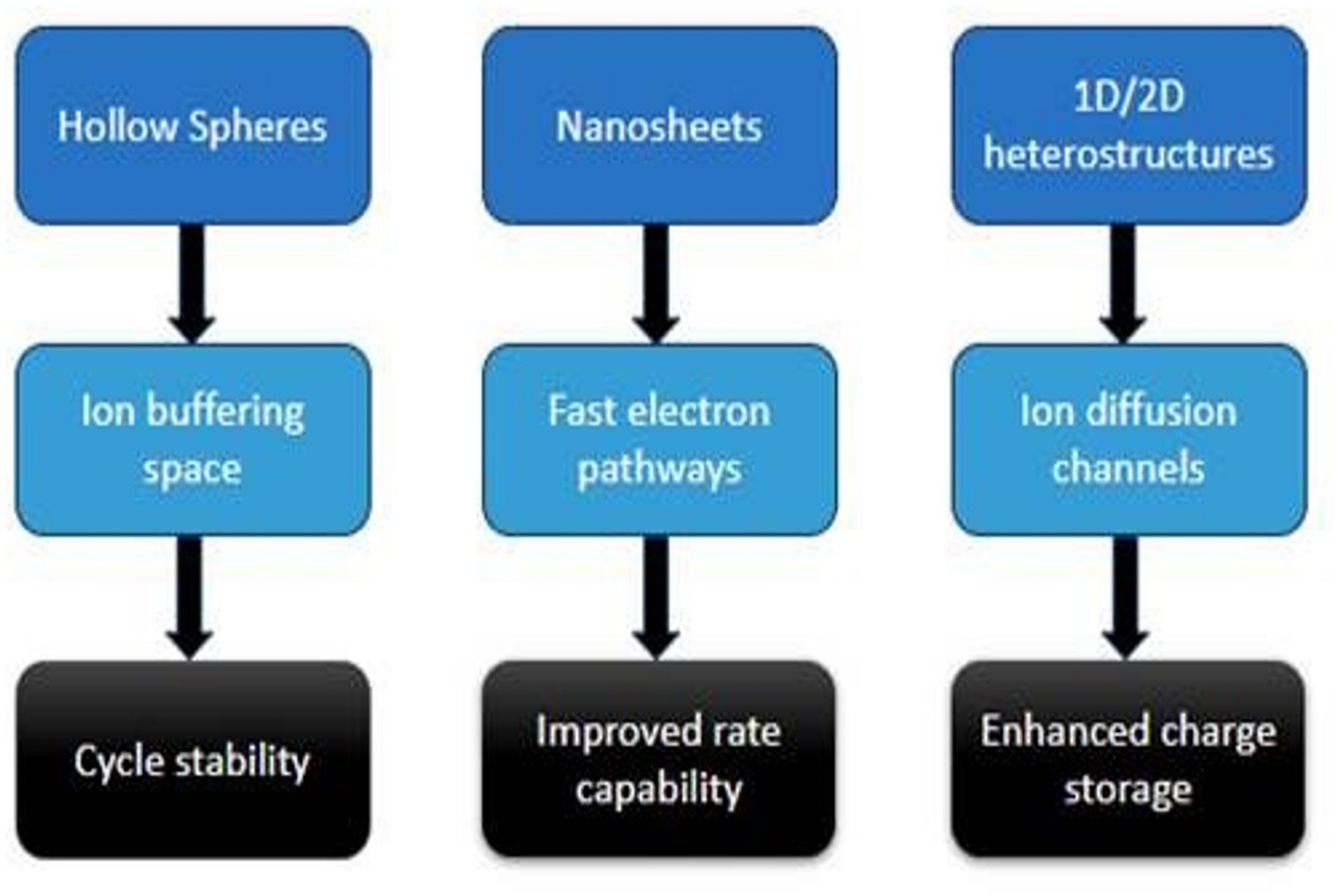
A sketch of the impacts of morphology on electrochemical performance.

Optimizing the performance of materials generated from TMCP requires morphological engineering. Hierarchical structures (hollow spheres, nanosheets, nanowires, hierarchical pores) provide high surface area and coupled ion diffusion channels, which improve charge storage. Higher capacitance and energy density are the results of improved electrolyte accessibility brought about by micro- and mesoporous structures. X-ray diffraction (XRD), scanning electron microscopy (SEM) and electrochemical impedance spectroscopy (EIS) techniques provide useful insights into material structure, morphology and electrochemical behaviour, which help guide the design of high-performance TMCP-derived materials. The sketch in figure 11 provides the structural diversity, which ranges from hollow microspheres and nanowires to complex 1D/2D heterostructures. These structures have a direct impact on ion diffusion kinetics, electron transport and electrode stability. Morphological control of TMCP-derived electrode materials is critical for determining electrochemical performance in supercapacitor applications [11,77–79].

**Figure 12.**
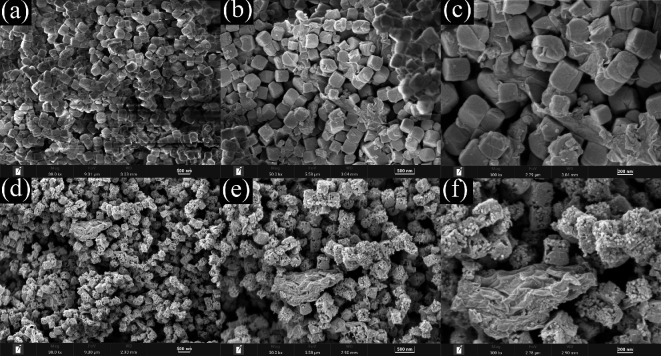
Co-CP/rGO (pre-calcination) FESEM images with magnifications of (a) ×30 000, (b) ×50 000 and (c) ×100 000, and Co_3_C/rGO (post-calcination) with magnifications of (d) ×30 000, (e) ×50 000 and (f) ×100 000. Reproduced with permission. © 2024 Trans Tech Publications Ltd [1].

A cyanide coordination polymer, Co_3_C/rGO, was investigated for use in supercapacitors. The study focuses on cobalt carbide nanocomposites that have a hollow cubic shape and exhibit low resistance (0.58 Ω) and high specific capacitance (486.6 F g^−^¹). The cubic morphology has a homogeneous size distribution in all samples of Co_3_C/rGO before and after calcination at 600°C as depicted in figure 12. The CP was eliminated after the calcination at this temperature. Furthermore, this method creates high porosity, making it easier to insert and retrieve electrolyte ions during charging and discharging. It emphasizes supercapacitor performance, hybrid integration and shape [1].

**Figure 13.**
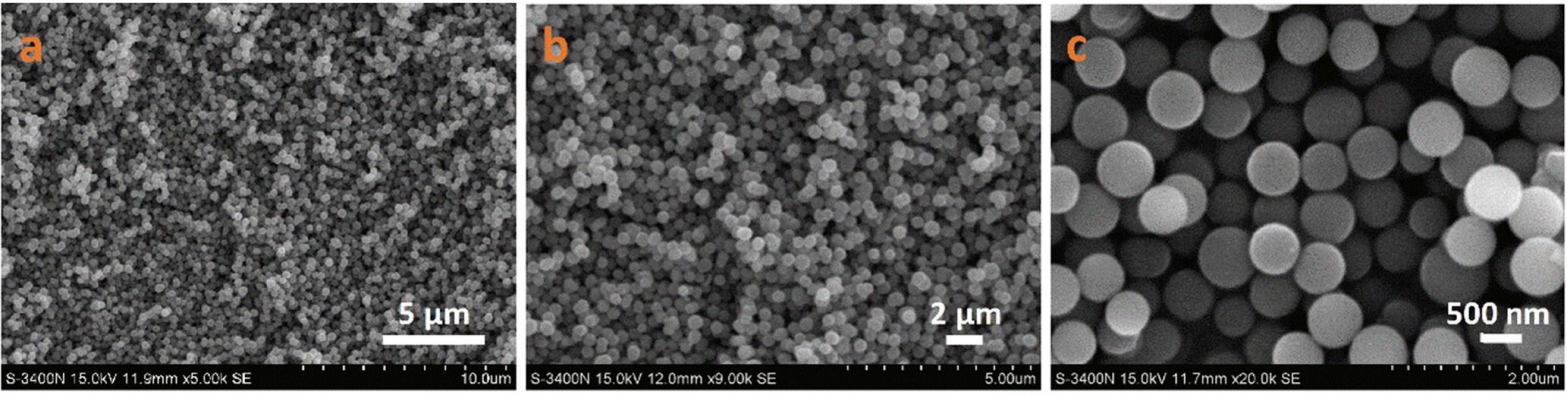
Mn–Ni–Co oxide microsphere SEM images (a–c). Reproduced with permission. © 2020 Elsevier [80].

Hollow microspheres with a yolk shell composed of Mn–Ni–Co oxide were prepared using a template-free solvothermal technique and post-heat treatment. The prepared Mn–Ni–Co oxides, as illustrated in figure 13a, are made up of numerous microspheres of very homogeneous size. The magnified SEM images (figure 13b,c) demonstrate that each rough-surfaced microsphere has a diameter of 500−600 nm. The obtained Mn–Ni–Co oxide electrode has a high specific capacitance (1173.4 F g^−1^ at 1 A g^−1^), excellent rate performance and good cycling stability (94% capacitance retention after 6000 cycles at 2 A g^−1^) due to its unique structural features, indicating its great potential for a variety of energy storage devices [80]. A hierarchical CoP@NiMn-P nanocomposite produced from TMCP is presented in a study for use in high-performance supercapacitors. Through hydrothermal synthesis, it integrates CoP nanowires and NiMn-P nanosheets to produce stable asymmetric supercapacitor (ASC) integration. After 5000 cycles at a current density of 10 A g^−1^, electrochemical analysis revealed that the specific capacitance reached 2162.2 F g^−1^ at 1 A g^−1^, with a high capacitance retention rate of 83.3%. The increased specific surface area and enhanced conductivity have been highlighted as reasons for the outstanding electrochemical performance. Furthermore, while it emphasizes performance optimization, hybrid system integration and morphological engineering, it makes no mention of sustainability or multifunctionality [81].

**Figure 14.**
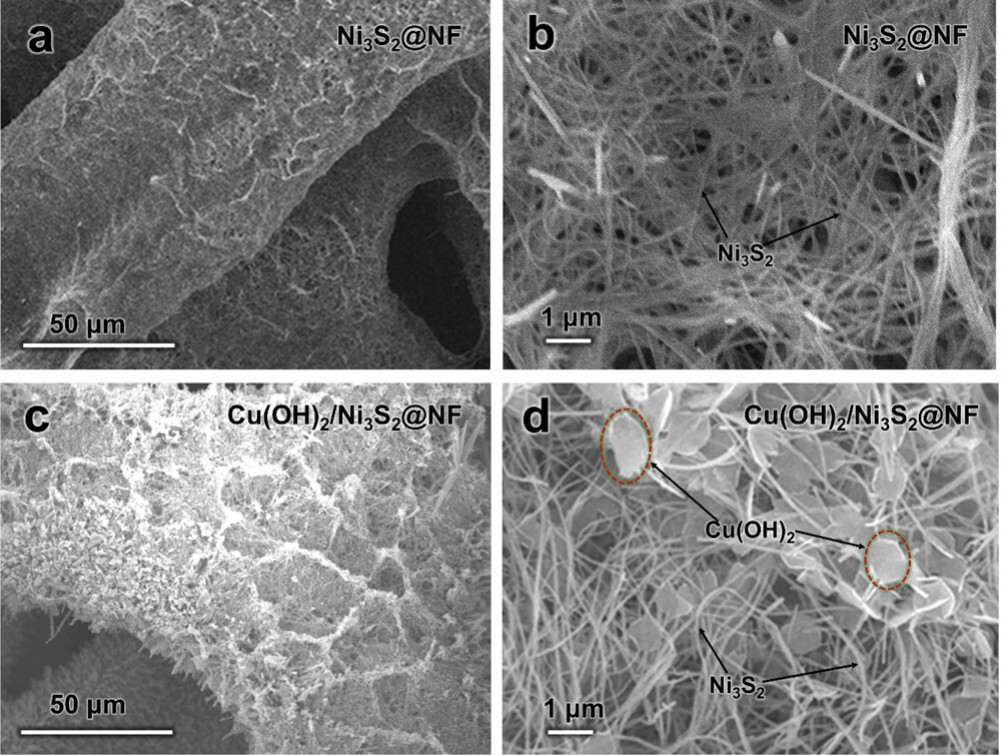
(a–d) Ni_3_S_2_@NF and Cu(OH)_2_/Ni_3_S_2_@NF SEM images at various magnifications. The dashed circles in (d) are Cu(OH)_2_ thin nanosheets. Reproduced with permission. © 2021 American Chemical Society [82].

**Figure 15.**
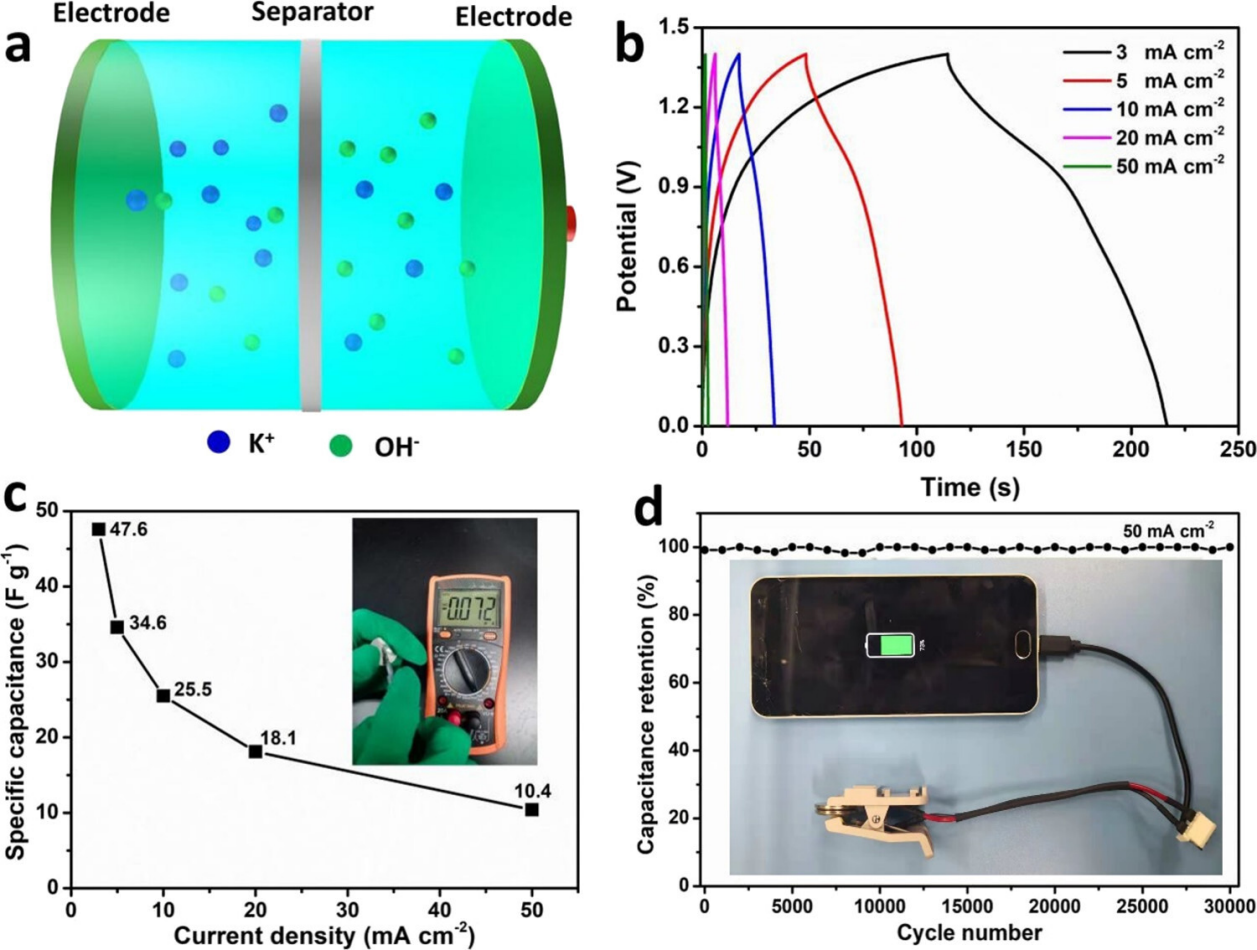
(a) Design of a coin-type cell including a two-electrode symmetric supercapacitor. (b) Cu(OH)_2_/Ni_3_S_2_@NF//Cu(OH)_2_/Ni_3_S_2_@NF GCD curves at different current densities. (c) Corresponding electrode capacitances at various current densities and digital images of a single coin-type cell that is linked to a multimeter (inset). (d) A mobile phone connected with a coin-type cell cycling for 30 000 cycles at 50 mA cm^–2^ (inset). Reproduced with permission. © 2021 American Chemical Society [82].

Cu(OH)_2_ nanosheets and Ni_3_S_2_ nanowire 2D/1D heterostructures are logically constructed into 3D porous network electrodes. The surface layer of nickel foam (NF) ligaments is chemically etched *in situ* using hydrothermal processes to create a 1D/2D heterostructure network. The Ni_3_S_2_@NF and Cu(OH)_2_/Ni_3_S_2_@NF electrodes’ low-magnification SEM images are shown in figure 14a,c. The samples exhibit a good preservation of the 3D porous structure of bare NF. In Cu(OH)_2_/Ni_3_S_2_@NF, the NF skeleton clearly has more folds than in Ni_3_S_2_@NF. High-magnification SEM pictures (figure 14b) show that the ligaments of the NF surface are uniformly covered in Ni_3_S_2_ nanowires. Following Cu(OH)_2_ growth, the 3D network of Ni_3_S_2_ nanowires (figure 14d) remained intact; in the meantime, a significant number of 2D nanosheets were dispersed throughout the nanowires to create 1D/2D heterostructures. The Cu(OH)_2_/Ni_3_S_2_@NF electrode exhibits exceptional stability and a high specific capacity of 1855 F g^−1^ at 2 mA cm^–2^ (figure 15). At 20 mA cm^–2^, the electrode retains more than 110% of its capacity after 35 000 cycles of charging and discharging. Strong mechanical bonds and robust electrical connections between Cu(OH)_2_/Ni_3_S_2_ active materials and the conductive NF, as well as highly accessible locations of the 3D network for electrolyte ions, are responsible for the improved performance. The unique 1D/2D heterostructure specifically helps to reduce structural pulverization during the ion insertion/desertion process [82].

From the discussion above, the importance of morphological engineering in improving the electrochemical performance of TMCP-derived materials used in supercapacitor applications was highlighted. Researchers have worked to significantly improved specific capacitance, ion diffusion rates and cycling stability by strategically designing hollow, hierarchical and hybrid nanostructures [79]. For instance, Co₃C/rGO nanocomposites, Mn–Ni–Co yolk–shell oxides, and CoP@NiMn-P hybrids have demonstrated high capacitances (over 2000 F g⁻¹) and excellent retention after thousands of cycles, owing to optimized shape and synergistic interactions among components. However, a comparative investigation finds major differences in synthesis techniques, electrode architectures and performance standards, emphasizing the lack of standardization across the field. There are still issues with comprehending the underlying redox mechanisms, attaining scalable and eco-friendly synthesis and integrating TMCP-derived materials into useful energy storage devices, despite significant advancements in the incorporation of conductive carbon materials and the use of advanced fabrication methods. The emphasis on performance improvement in current research also frequently ignores sustainability measures, environmental effect and multifunctionality. The next generation of high-performance, sustainable supercapacitor technologies must be paved with a more comprehensive strategy that integrates life-cycle analysis, green chemistry and the investigation of under-represented metal systems with electrochemical optimization.

### 4.2. Pseudocapacitance and faradaic reactions in TMCPs

**Figure 16.**
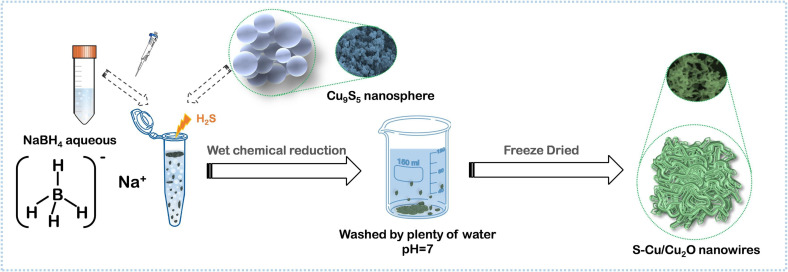
S-Cu/Cu_2_O sample synthesis process. Reproduced with permission. © 2024 Elsevier [86].

The unique properties of transition metals such as high electronic conductivity, partially filled d-orbitals, and diverse oxidation states enable robust redox reactions and effective charge transfer in CP-derived supercapacitors. Incorporation of transition metals improves the pseudocapacitive behaviour of CP-derived supercapacitors compared with traditional double-layer capacitors, resulting in higher energy densities. TMCPs are a promising family of materials due to their modular architectures, excellent tunability and potential for a wide range of energy storage applications [11,20,83]. Nickel-based CP-derived materials generally store energy via pseudocapacitive methods, including reversible redox reactions of nickel ions. Electrochemical experiments, for example, show that converting Ni(II) to Ni(III) greatly increases charge storage capacity [84]. Through the phase transition from metal sulfide to metal selenide, an amorphous/crystalline heterostructure was created in a research work by selenizing the self-sacrificial template NiMnS to produce an amorphous Mn/polycrystalline Ni_0.85_Se–NiSe_2_ heterophase. During charging and discharging, the amorphous/polycrystalline heterophase and complementary multi-components worked together to enhance electron/ion-transport channels and reveal many active sites, which sped up electron/ion transfer and faradaic reaction kinetics. The ideal NiMnSe demonstrated a high specific charge (1389.1 C g^−1^ at 1 A g^−1^), a fair rate capability, and an outstanding longevity (88.9% retention), as anticipated. The manufactured NiMnSe//activated carbon device also demonstrated a long cycle life and an energy density of 48.0 Wh kg^−1^ at 800 W kg^−1^, which suggests that it could be used in electrochemical energy-storage devices and other practical applications [85]. Cu-based CPs can be transformed into copper oxides or sulfides using thermal or chemical methods. Strategies such as doping with nitrogen or sulfur have been utilized to enhance the conductivity and electrochemical activity of Cu-based CP-derived materials. Luo *et al.* used a simple *in situ* wet chemical reduction approach to produce an S-modified Cu/Cu_2_O for hybrid capacitors as illustrated in figure 16. The S can influence the electrical structure, promoting electron transfer during the charge/discharge process. A high capacity of 669 mAh g^−1^ at 1 A g^−1^ was achieved [86].

Cu-CPs and their derivatives have pseudocapacitive behaviour due to reversible redox processes involving copper ions. CuO/Cu_2_O nanostructures made from Cu-CPs are advantageous for attaining high specific capacitance [86,87]. The reversible redox reactions of iron ions give rise to the pseudocapacitive behaviour of Fe-CPs. The transition between the Fe(II) and Fe(III) states plays a major role in charge storage. Furthermore, the open structures of Fe-CPs give electrolyte ions plenty of room, improving the effectiveness of charge storage and ion movement [32,88]. Fe-CP-derived materials have high specific capacitances. Energy and power densities are competitive when high specific capacitance and quick charge/discharge capabilities are combined. For instance, an energy density of 38 Wh kg⁻¹ was reported at a power density of 8 kW kg⁻¹ [32]. The electrochemical performance of Zn-CPs is mostly due to pseudocapacitive behaviour caused by reversible redox reactions of zinc ions. As highlighted, redox shifts between Zn(II) and Zn(III) states contribute significantly to charge storage [49,89–91].

### 4.3. Hybrid systems and multifunctional applications

#### 4.3.1. Hybrid materials and carbon composites

**Figure 17.**
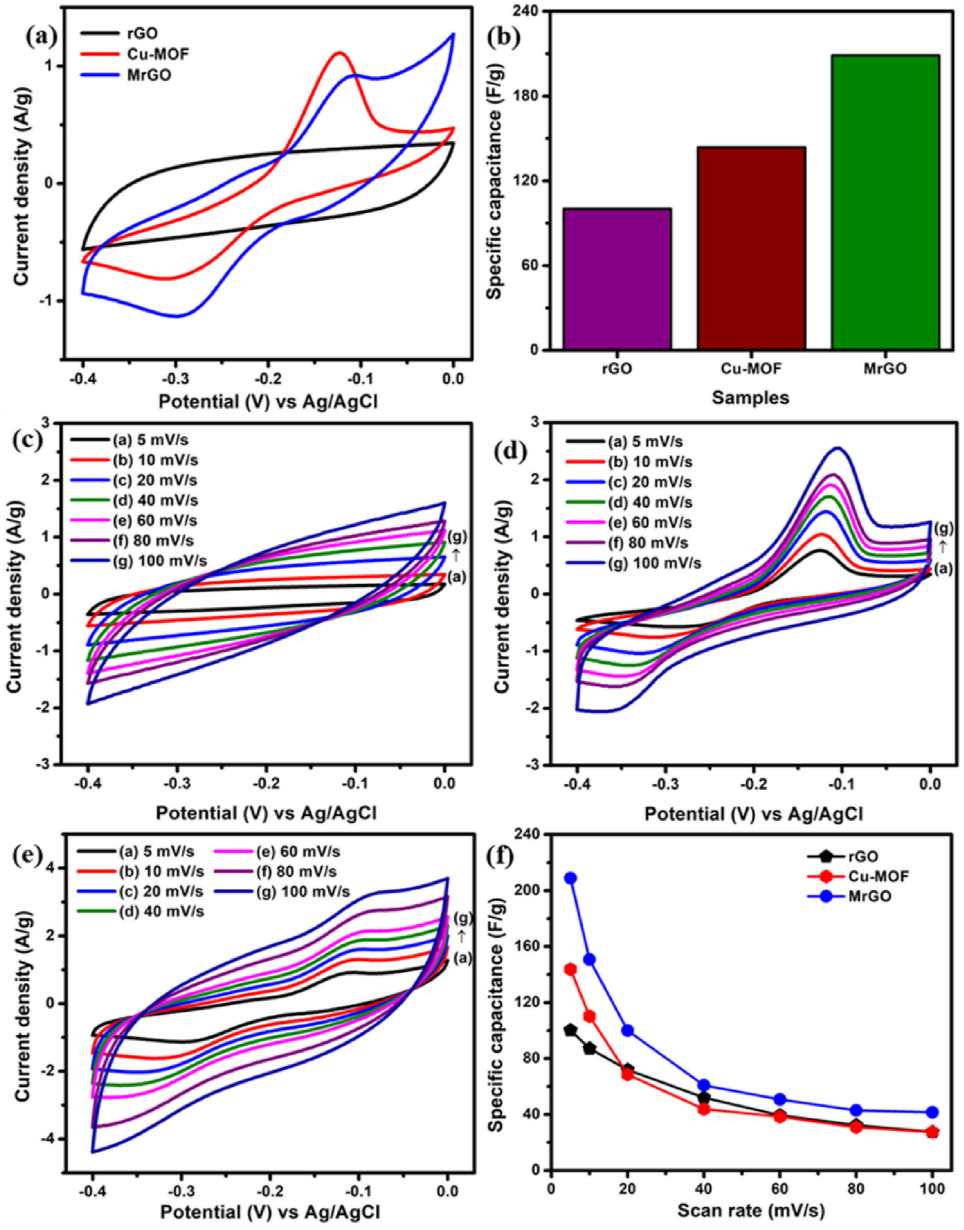
(a) CV curves of rGO, Cu-MOF and MrGO at 5  mV s^−1^. (b) Comparison of specific capacitance for various negative samples at 5  mV s^−1^. The CV curves of (c) rGO, (d) Cu-MOF and (e) MrGO at varied scan rates (5–100 mV s^−1^) and (f) the specific capacitance of rGO, Cu-MOF and MrGO electrodes at various scan rates. Reproduced with permission. © 2021 Elsevier [92].

When conductive materials like graphene, carbon nanotubes (CNTs) or activated carbon are hybridized, the conductivity and structural stability of electrodes formed from TMCPs is greatly enhanced. TMCPs’ redox activity is enhanced by graphene’s high conductivity and flexibility, which enhances performance synergistically [55]. For example, Yazar describes the improved performance of Cu-CP/graphene composites in supercapacitors. Copper-based electrodes with S-, N- and Cl-doped GO independently have been synthesized using the galvanostatic electrodeposition method for supercapacitors. The electrodes provide the ideal surface to increase the specific surface area and provide electrically active fields for the Faraday reaction. At 5 mV s^−1^, the Cl-GO@CuO/Cu_2_O electrode has a maximum capacity of 577 F g^−1^. Furthermore, a maximum energy density of 25.3 Wh kg^−1^ and maximum power density of 700 W kg^−1^ were attained by the Cl-GO@CuO/Cu_2_O symmetric supercapacitor. The device is stable for up to 2000 cycles with a capacitance retention of 97.07% [87]. Cu-MOF and rGO were combined to produce the MrGO composite. The VrGO/MrGO ASC device has an exceptional specific capacitance of 483.9 F g^−1^ and exhibited an outstanding specific energy of 31.2 Wh kg^−1^. Figure 17a–f illustrates the CV curves and specific capacitance at various scan rate [92].

**Figure 18.**
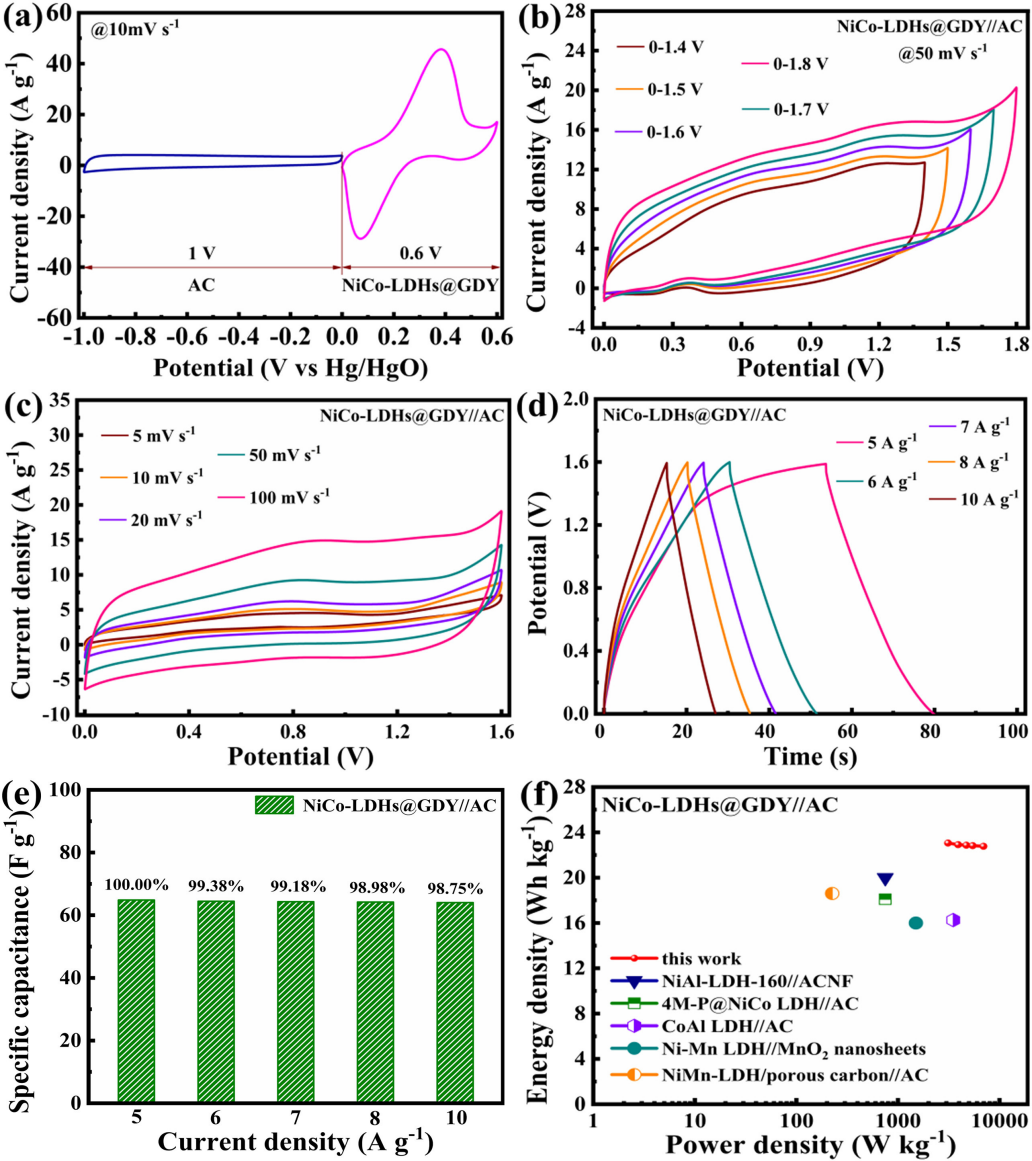
Evaluation of the electrochemical performance of NiCo-LDHs@GDY and AC (a–f). Reproduced with permission. © 2023 Elsevier [93].

Furthermore, adding nickel into composites with carbonaceous materials (graphene, carbon nanotubes) or metal oxides (MnO_2_, Co_3_O_4_) has resulted in higher energy and power density. For instance, the activated carbon-assembled NiCo-LDHs@GDY//AC ASC exhibits a high power density of 3108.96 W kg^−1^ and an energy density of 23.06 Wh kg^−1^. After 5200 charge–discharge cycles at a current density of 10 A g^−1^, the charge storage capacity stays at 89.53%. These findings demonstrate that the NiCo-LDHs@GDY electrode material (figure 18a–f) has significant application potential and can improve the properties of high-performance supercapacitors in energy storage applications [93].

**Figure 19.**
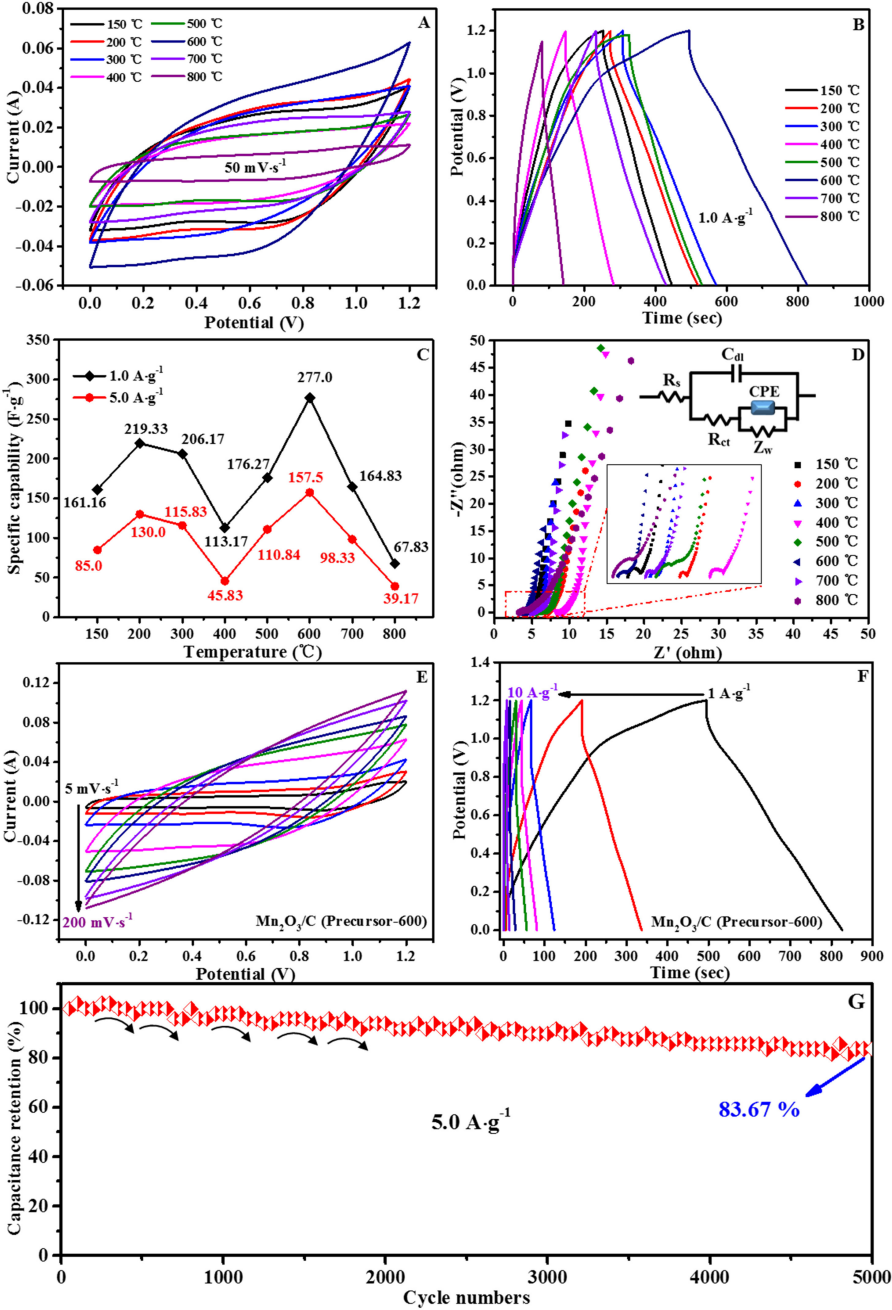
Electrochemical characteristics of precursors and derivatives in a three-electrode setup at 600°C. (a) CV curves; (b) CP profiles; (c) specific capability at 1.0 A g^−1^ and 5.0 A g^−1^ currents; (d) Nyquist plots (the corresponding circuit is shown in the inset); (e) CV curves; (f) GCP profiles; and (g) cycle stability. Reproduced with permission. © 2021 Elsevier [48].

Stable cycle performance is ensured using carbon nanotube composites, which increase mechanical strength and electron transfer efficiency. The hierarchical porosity of Co-CPs promotes efficient ion transport and electron transfer, which improves their performance [94]. A study by Li *et al.* uses a plasma modification technique to control the amounts of Co_4_N/CoN heterostructures and the surface morphology of MOF materials, yielding a Co_4_N/CoN@C-P material. This material combines the benefits of porous MOFs with N-doped carbon structures, with the Co_4_N/CoN heterostructure speeding charge transfer at the crystal interface and increasing electrical conductivity. The Co_4_N/CoN@C-P has a high specific capacitance of 346.2 F g^−1^ at 1 A g^−1^. Co_4_N/CoN@C-P has a specific capacitance of 335.6 F g^−1^ at 10 A g^−1^, roughly 96.9% of that at 1 A g^−1^, showing excellent rate capability. Furthermore, the capacitance retention stays at 100% even after 1000 cycles, implying exceptional cycling stability. The excellent electrochemical performance of this new composite material is due to the nanoscale plasma engineering and logical structure of the transition metal nitride heterostructures [95]. Fe-CP/reduced graphene oxide composites were fabricated and claimed to have much better cycling stability and rate capability. Fe-CP/graphene composites performed better than pristine Fe-CPs. By forming hierarchical porous structures inside Fe-CPs, specific capacitance is improved and ion diffusion is facilitated. Template-assisted synthesis techniques have produced such structures successfully [32,43]. An *in situ* approach was used to synthesize nanowire/carbon sphere composite materials, and the morphological structure and crystal phase evolution of Mn_*x*_O_*y*_ were examined with the annealing temperature as a variable. After annealing the precursors at 600°C, the rod-like nanoarray Mn_2_O_3_/C composites exhibit excellent specific capacitance and rate performance throughout an extensive potential window of 0–1.2 V (with a remarkable specific capacitance of 277.0 F g^−1^ at a current density of 1.0 A g^−1^). Figure 19 shows the electrochemical characteristics of precursors and derivatives at 600°C in a three-electrode arrangement. The Mn_2_O_3_/C//Na_2_SO_4_//Mn_2_O_3_/C symmetrical supercapacitor device as assembled shows a maximum energy density of 80.35 Wh kg^−1^ at a power density of 500 W kg^−1^, which can be used as promising storage materials for the information era [48].

**Figure 20.**
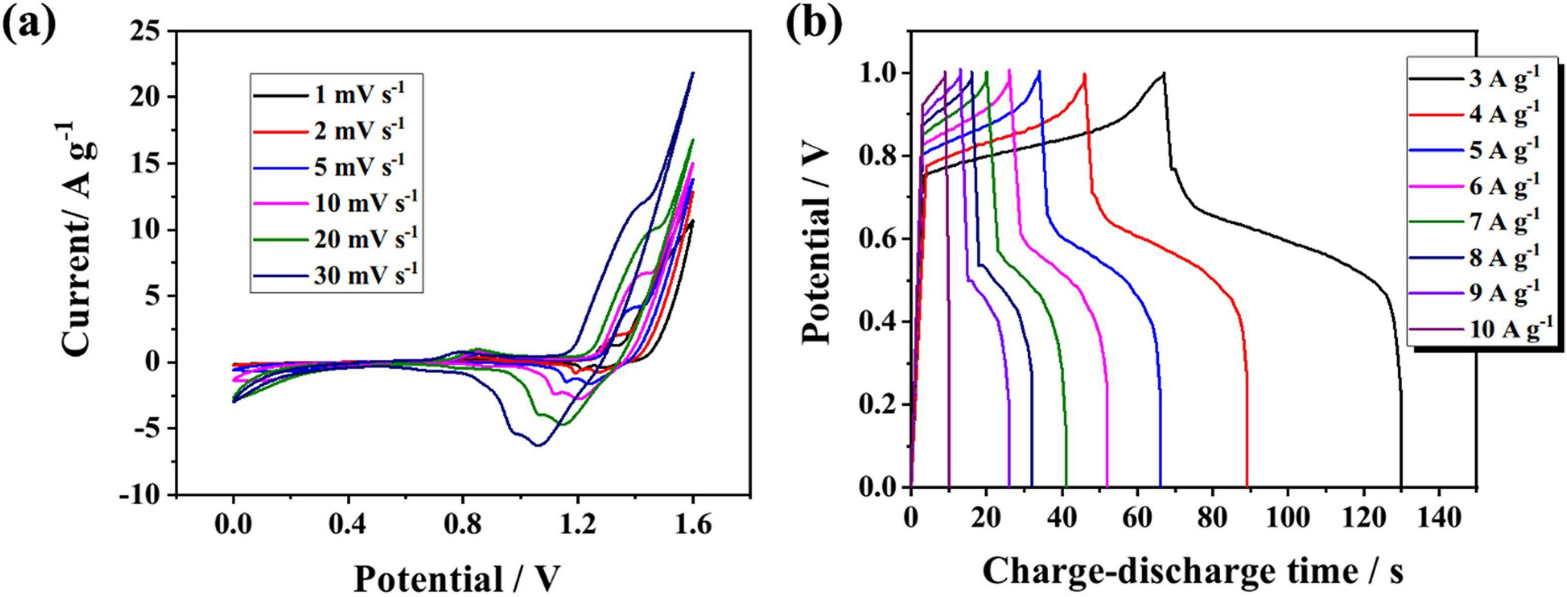
(a) CV curves of HPZC-4//AC ASC at various scan rates in the range 1 mV s^−1^ to 30 mV s^−1^. (b) GCD curves of HPZC-4//AC ASC at various current densities in the range 3 A g^−1^ to 10 A g^−1^. Reproduced with permission. © 2020 Elsevier [96].

A honeycomb-like zinc carbo-diimide/graphitized carbon nitride (ZnNCN/g-C_3_N_4_) based nanocomposite (NC) electrode was developed by Shen *et al.*, using a simple heat treatment technique. It yielded a capacitance of 5975 F g^−1^ at 1 A g^−1^ in 3 M KOH with an 87.9% retention capacity over 3000 cycles at 7 A g^−1^. The typical CV curves for HPZC-4//AC ASC at different scan speeds in the 0−1.6 V range are displayed in figure 20a and the pertinent GCD curves for the 3−10 A g^−1^ and 0−1 V potential windows are displayed in figure 20b [96]. Furthermore, a ZnNCN/g-C_3_N_4_//AC ASC device was produced. At 3 A g^−1^ current density in 3 M KOH, the ASC showed a capacitance, energy density and power density of 779 F g^−1^, 213 Wh kg^−1^ and 7839 W kg^−1^, respectively. Additionally, following 2000 charge–discharge cycles at 7 A g^−1^, the ASC demonstrated outstanding cyclic stability of 87.6% [97].

#### 4.3.2. Synergic effects in bimetallic and trimetallic systems

**Figure 21.**
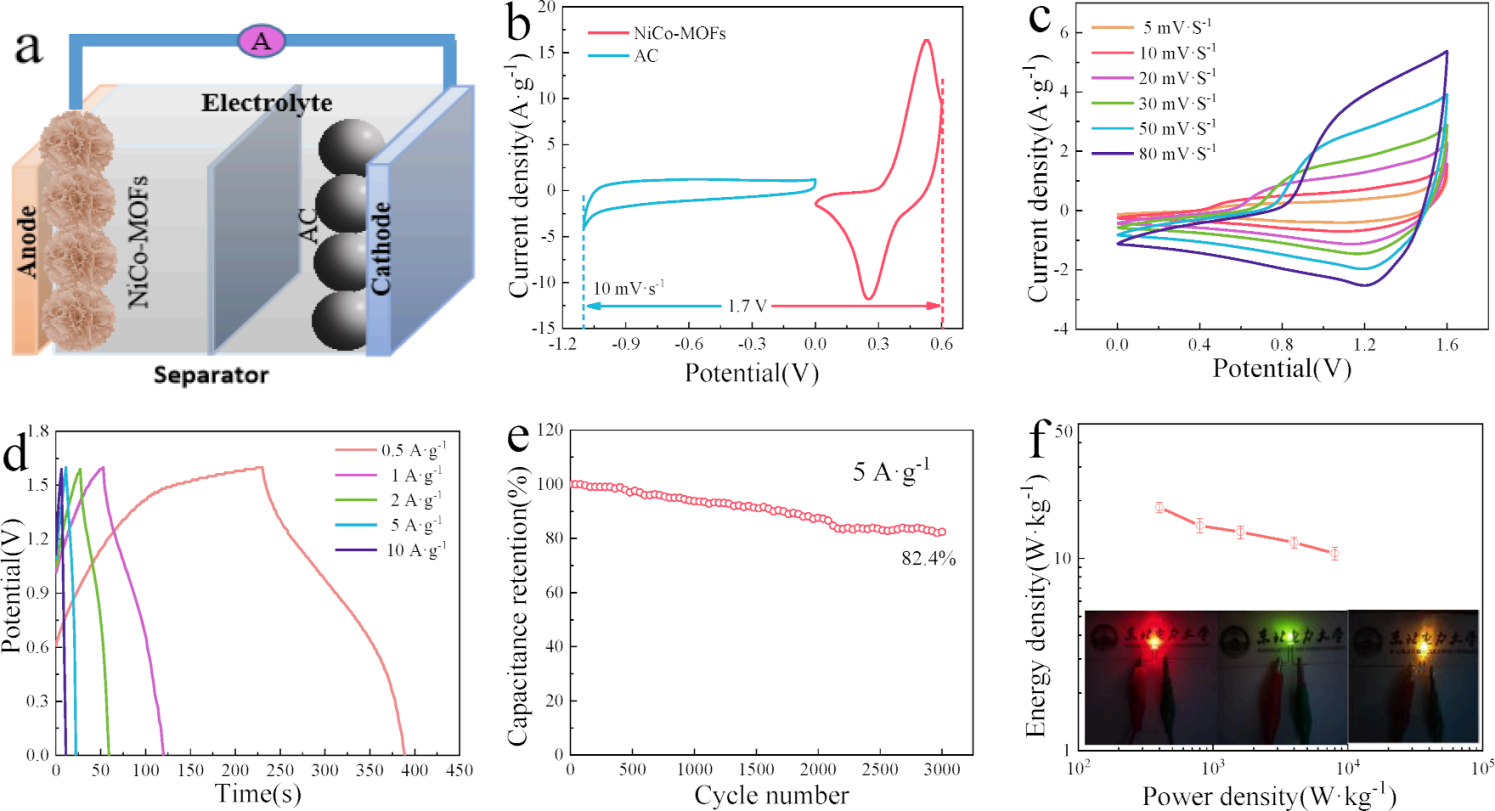
The embedded diagram displays the following: (a) the NiCo-MOFs//AC HSC setup diagram; (b) the NiCo-MOFs and AC CV curves at 10 mV s^−1^; (c) the NiCo-MOFs//AC HSC CV curves at 5−80 mV s^−1^; (d) the GCD curves of the NiCo-MOFs//AC HSCs at 0.5−10 A g^−1^; (e) the NiCo−MOFs//AC HSC cycling life at 5 A g^−1^; (f) the Ragone plot of the NiCo-MOFs//AC HSCs and lit LEDs. Reproduced with permission. © 2021 MDPI [98].

TMCPs are a promising class of materials because of their modular structures, high tunability and potential for a variety of energy storage uses. The structural design of CP precursors has prompted the emergence of innovative chemistries and architectures such as monometallic, bimetallic and even polymetallic systems, potentially improving the electrochemical performance of the end products. There are distinct benefits to each system [11,20,83]. The addition of nickel to bimetallic CPs, particularly MOFs, such as Ni-Co, has resulted in materials with higher redox activity and cycling stability. NiCo-MOFs have a specific capacitance of 882 F g^−1^ at 0.5 A g^−1^ and can retain a cycle life of 90.1% after 3000 charge–discharge cycles at 5 A g^−1^. Furthermore, the NiCo-MOFs//AC HSCs, made of NiCo-MOFs and activated carbon (AC), attained a maximum energy density of 18.33 Wh kg^−1^ at a power density of 400 W kg^−1^, and demonstrated good cycle life (82.4% after 3000 cycles). These exceptional electrochemical capabilities showcased in figure 21a–f can be attributed to the synergistic action of metal ions, the optimized conductivity, and the unique layered stacked floral structure, which provides a smooth transmission channel for electrons/ions [98].

**Figure 22.**
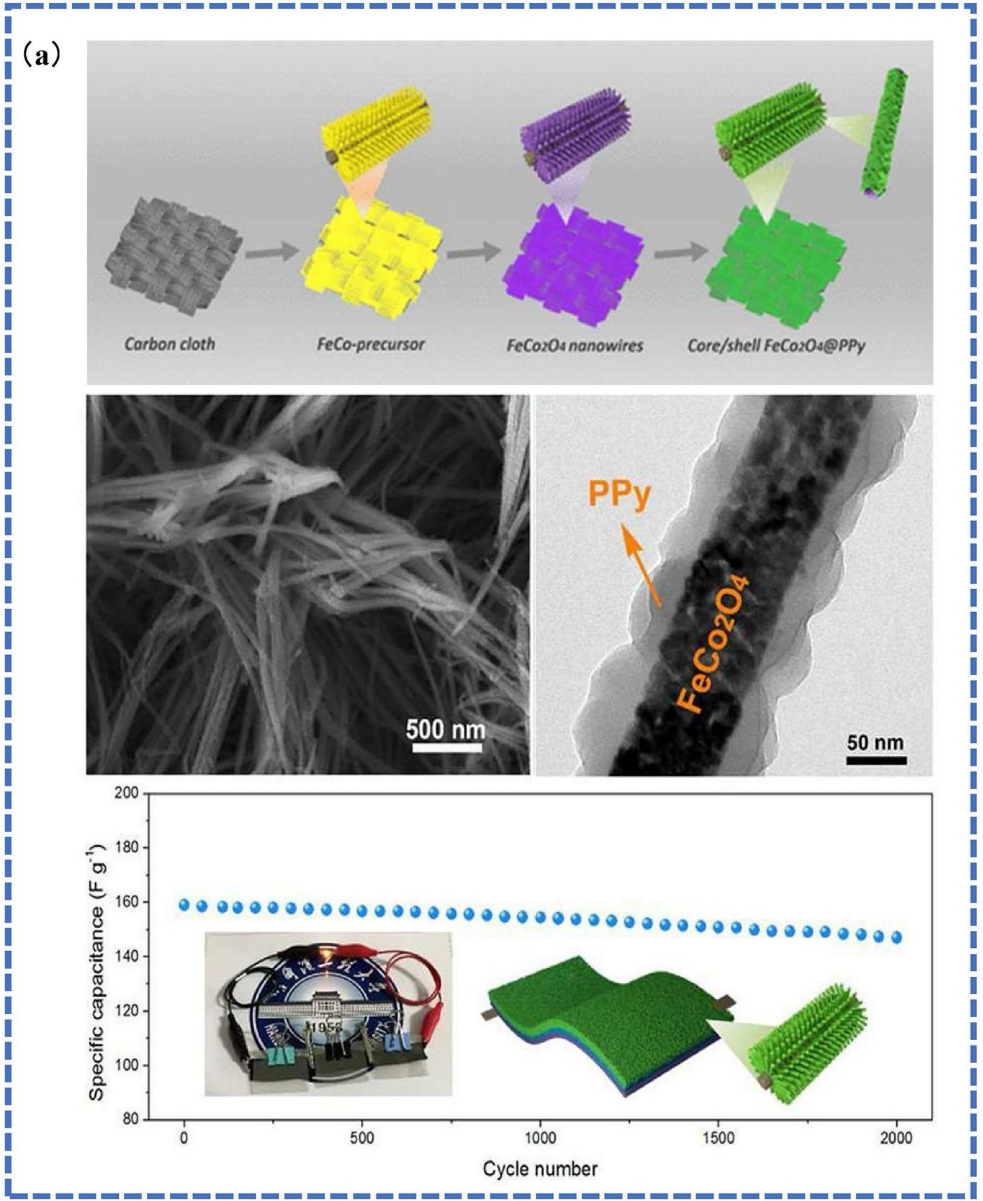
Schematic of fabrication process of FeCo_2_O_4_@PPy. Reproduced with permission. © 2023 Elsevier [32].

Li *et al.* reported the synergistic effects of bimetallic CP systems, such as Co–Ni and Co–Fe CPs, leading to increased redox activity and charge storage capacity. The study proposes a new approach for manufacturing high-specific-capacity nickel–cobalt-based composite materials through nanoscale structure control, with a stable and efficient strategy having extensive application potential [38]. The performance of Cu-based CPs can be improved through structural changes and functionalization. It has been demonstrated that adding a second metal, such as nickel or cobalt, to the Cu-based CP framework increases redox activity and electrical conductivity. Cu–Ni bimetallic CPs have synergistic benefits on specific capacitance and cycling stability. Cu–Co and Cu–Fe CPs are examples of bimetallic and polymetallic systems that use the combined effects of many metals to improve cycling stability and charge storage. Cu–Co CPs perform better than their monometallic equivalents [42,99,100]. Fe–CP materials have demonstrated capacitance retention above 90% after 10 000 cycles and the synergistic interactions between the two metal centres in bimetallic Fe-based CPs result in increased redox activity. Figure 22 shows a schematic illustration of the fabrication process of FeCo_2_O_4_@PPy. The optimized FeCo_2_O_4_@PPy electrodes have an excellent specific capacitance of 2269 F g⁻¹. Furthermore, they displayed excellent cycling stability with 90.2% capacitance retention after 5000 cycles, underlining their performance. Fe–Co bimetallic CPs performed better than their monometallic counterparts. Fe-based CPs can be thermally converted into metal oxides or sulfides, resulting in greatly improved electrical conductivity and pseudocapacitive characteristics. Fe₃O₄-based materials generated from Fe-based CPs demonstrated high specific capacitance and outstanding rate capability [32,42,88]. Bimetallic Mn-based CPs, such as Mn–Ni and Mn–Co systems, exhibit synergistic effects that enhance redox activity and stability. For example, it was reported that Mn–Ni CPs displayed superior specific capacitance due to enhanced electron transport, and it was also shown that MnO₂ derived from Mn-based CPs achieved a specific capacitance of 1800 F g⁻¹ [47,101].

**Figure 23.**
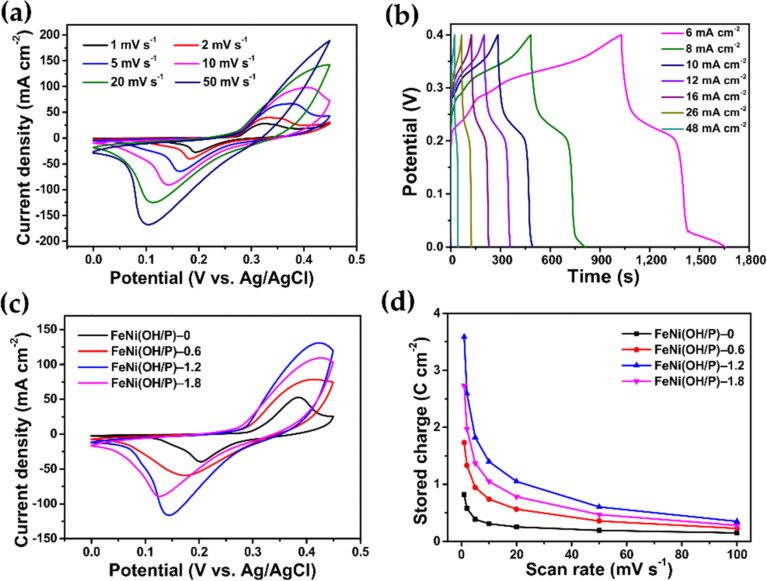
(a) CV curves at various scan rates and (b) GCD curves at varying current densities for the FeNi(OH/P)-1.2 electrode. Comparison of (c) CV curves and (d) rate performance for FeNi(OH/P) electrodes with varying NaH_2_PO concentrations. Reproduced with permission. © 2022 MDPI [102].

A simple two-step process was used to produce a self-supported FeNi(OH/P) nanosheet array that resembles a nanohoneycomb. The FeNi(OH/P) electrode achieved saturation phosphorization and nanostructure optimization at the same time by modulating the NaH_2_PO_2_ concentration. The constructed FeNi(OH/P) electrode achieved a significantly improved specific capacity of 3.6 C cm^−2^ (about 408.3 mAh g^−1^) at 1 mV s^−1^ with cyclic stability (72.0 %) after 10 000 cycles. An all-solid-state hybrid supercapacitor device with FeNi(OH/P) as the cathode electrode and PPy/C as the anode electrode achieved a high capacity of 1.9 C cm^−2^ at 7 mA cm^−2^, a maximum energy density of 45 Wh kg^−1^ shown in figure 23, and excellent cyclic stability of 118.5% after 2000 cycles after optimizing the electrolyte. These good qualities imply that the FeNi(OH/P) nanosheet array has excellent prospects as an advanced electrode material for HSCs [102].

#### 4.3.3. Integration with advanced materials

**Figure 24.**
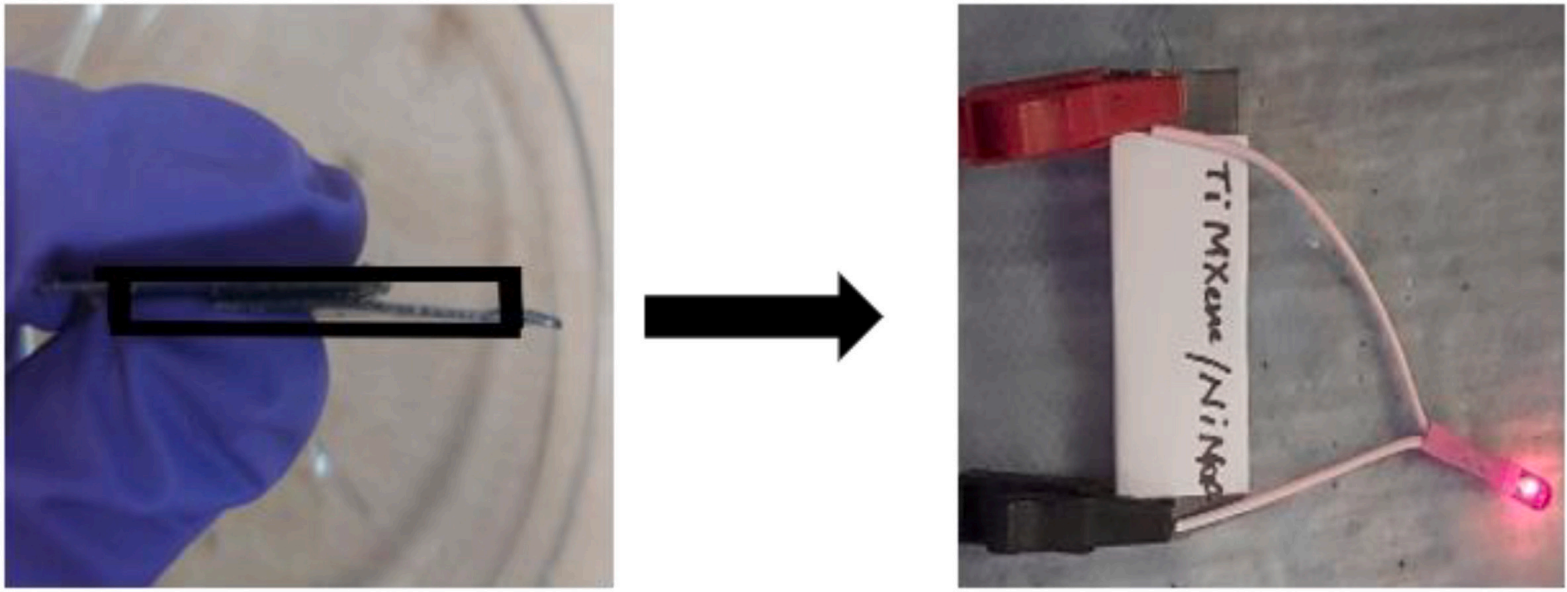
Symmetrical device (two-electrode assembly with PVA in H_2_SO_4_ electrolyte). Reproduced with permission. © 2024 Elsevier [104].

The integration of advanced materials, such as MXenes and graphene, enhances the performance of TMCP-based devices [103]. A study has investigated a composite (Ti_3_C_2_T_*x*_ /Ni-MOF) of titanium carbide MXene (Ti_3_C_2_T_*x*_) and nickel-based MOF (Ni–NH_2_BDC MOF) for supercapacitor application. The rationale for the use of the composite is that, by preventing the restacking and oxidation of MXene sheets, the composite helped to induce stability in the overall system. The electrochemical properties of these Ti_3_C_2_T_*x*_ nanosheets intercalated with MOFs showed a notable improvement in capacitance and power density both before and after composite formation. Using a polymer-based gel electrolyte (polyvinyl alcohol in 1 M H_2_SO_4_) and two electrodes of comparable weight, the symmetric supercapacitor device was set up (figure 24). With an energy density of 19.4 Wh kg^−1^ and a power density of 331.8 W kg^−1^, the device offered a potential window of 0−2.0 V and a specific capacitance of 139.4 F g^−1^ at a current density of 1 A g^−1^. Even after 5000 cycles of charging and discharging, a 95% retention of the capacitance was noted. Future energy storage applications may find the synthesized composite to be an appropriate electrode material, as confirmed by the observed response [104].

**Figure 25.**
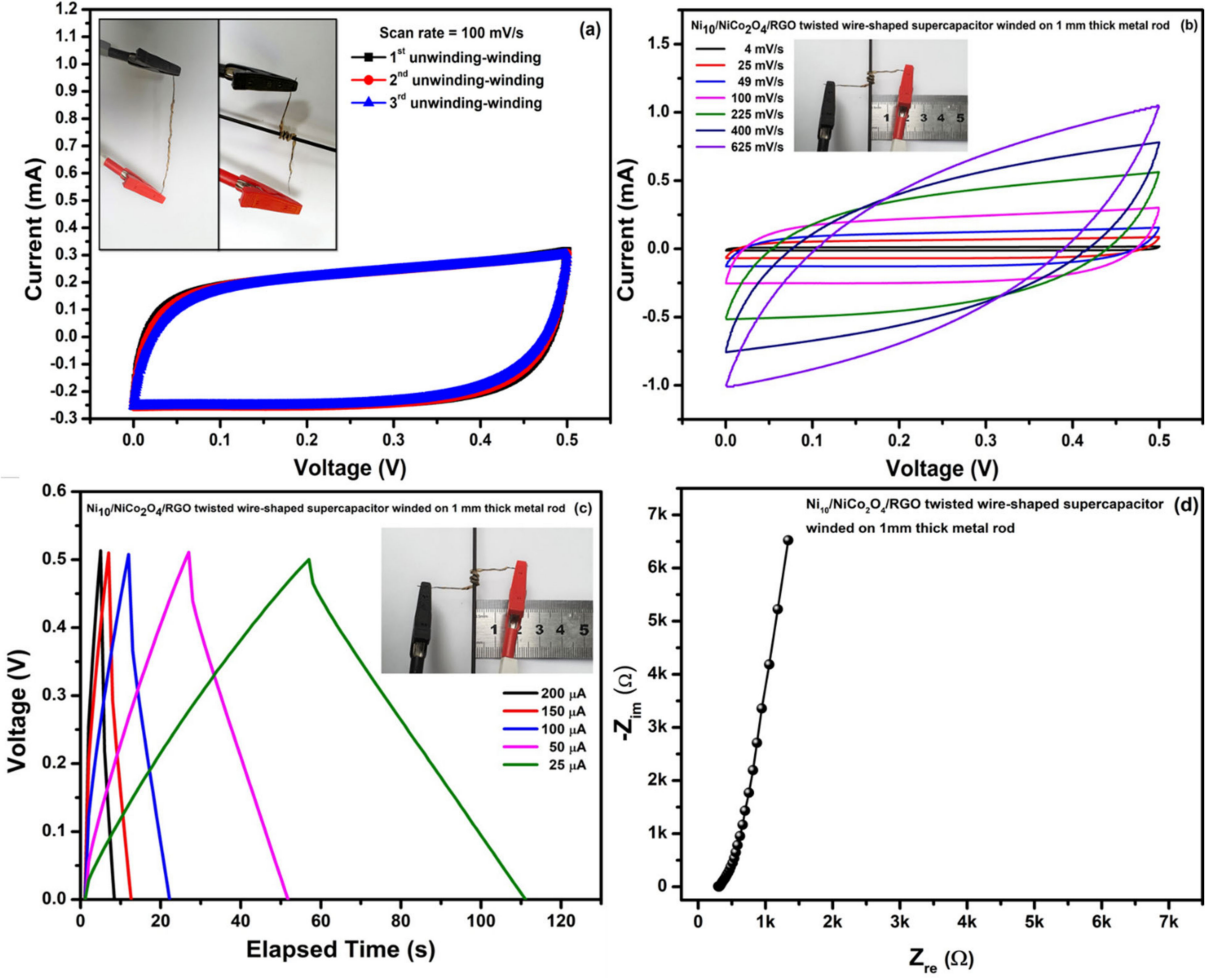
(a) CV curves for Ni_10_/NCO/RGO twisted WSC unwinding–winding sequences at 100 mV s^−1^ scan rate; (b) CV curves for WSC in wound state at different scan rates; (c) GCD curves for WSC in wound state at different applied currents; and (d) EIS study (Nyquist plot) for WSC in wound state at 100 kHz−0.01 Hz under 0.01 V. Reproduced with permission. © 2021 MDPI [107].

By using freeze-drying assisted mechanical pressing, a 3D porous compact 1D/2D Fe_2_O_3_/MXene aerogel film electrode with improved electrochemical performance was developed. After adding 1D α-Fe_2_O_3_ nanorods, the resulting Fe_2_O_3_/MXene aerogel film electrode exhibits an improved specific capacitance of 182 F g^−1^ (691 mF cm^−2^) at a current density of 1 A g^−1^ in 3 M H_2_SO_4_ electrolyte, as well as an 81.74% capacitance retention after 10 000 charge–discharge cycles. Additionally, the volumetric capacitance of the composite aerogel film (150 F cm^−3^) is significantly increased, making it 2.68 times that of the pure MXene aerogel film (56 F cm^−3^). Additionally, at a power density of 119.04 µW cm^−2^, the manufactured all-solid-state symmetric supercapacitor (SSSC) maintains a superior areal energy density of 3.61 µWh cm^−2^. An advanced method of making an MXene-based supercapacitor electrode is offered by this rapidly growing 3D porous, binder-free, freestanding aerogel film [105]. Through the annealing of CoNi-cyanide bridged coordination polymers (CoNi-CP) in a nitrogen atmosphere, a study developed hybrids of CoNi-carbide (CoNi-C) and reduced graphene oxide (rGO). Excellent electrochemical performance was demonstrated by the resultant CoNi-C/rGO hybrids, outperforming the CoNi-C and rGO components separately. According to the hybrids, their electroactive surface area was 130.87 m^2^ g^−1^ and their specific capacitance was 1177 F g^−1^. To get the maximum specific capacitance, the CoNi-C/rGO ratio was optimized. In addition, a coin cell was built using rGO as the negative electrode and CoNi-C/rGO-2 as the positive electrode. It performed exceptionally well, exhibiting an 84% capacitive retention after 8000 charging cycles and an energy density of 31.6 Wh kg^−1^ at a power density of 750 W kg^−1^. Considering prospective applications in a range of devices, this research offers important insights into the design and development of high-performance electrode materials for energy storage [106]. Shewale and Yun synthesized surface-modified Ni wire/NiCo_2_O_4_/reduced graphene oxide (Ni/NCO/RGO) electrodes for use in wire-shaped supercapacitors. These electrodes were formed using a mix of simple solvothermal and hydrothermal deposition techniques. A thorough investigation was conducted into how Ni wire etching affected the electrochemical, surface morphological and microstructural characteristics of Ni/NCO/RGO electrodes. The electrode made from Ni wire etched for 10 min, or Ni_10_/NCO/RGO, has the lowest initial equivalent resistance (1.68 Ω) and a good rate capability with a volumetric capacitance (2.64 F cm^−3^) and areal capacitance (25.3 mF cm^−1^). This is due to the enhanced hybrid nanostructure and the synergistic effect between spinel-NiCo_2_O_4_ hollow microspheres and RGO nanoflakes. Furthermore, it was discovered that the volumetric specific capacitance, which was determined by considering simply the volume of active material, was as high as 253 F cm^−1^. Comparing the Ni_10_/NCO/RGO electrode to other electrodes that had the best electrochemical performance, it is shown that the diffusion-controlled process associated with faradaic volume processes (battery type) made a substantial contribution to the surface-controlled process. Moreover, two optimal electrodes were assembled in a twisted structure using gel electrolyte to create the wire-shaped supercapacitor (WSC), which demonstrated (figure 25) an energy density of 10 μWh cm^−3^ (54 mWh kg^−1^) and a power density of 4.95 mW cm^−3^ (27 W kg^−1^) at 200 μA. Lastly, it was successfully shown that WSCs are repeatable, flexible and scalable at different device lengths and bending angles [107]. Cobalt phosphide and cobalt nanoparticles embedded in nitrogen-doped nanoporous carbon (CoP-CoNC/CC) were effectively synthesized by a simple precipitation process followed by pyrolysis (phosphorization). Then, for supercapacitor applications, CoP-CoNC/CC was used as the electrode. The resulting CoP-CoNC/CC was notable for having a large surface area and adjustable porosity. Improved electrochemical performance was attained with a specific capacitance of 975 F g^−1^ at 1 mA cm^−2^ in a 2 M KOH electrolyte, based on the advantages of the CoP in CoNC/CC. In addition to having higher cyclic stability, the constructed hybrid supercapacitor with activated carbon (AC) as the negative electrode and CoP-CoNC/CC as the positive electrode demonstrated a specific capacitance of 144 F g^−1^ and a specific energy of 39.2 W h kg^−1^ at 1960 W kg^−1^ specific power. The synergistic interaction of CoP, Co metal and the nitrogen-doped nanoporous carbon in 3D carbon cloth (CC) is responsible for the improved performance. Because of these superior qualities, CoP-CoNC/CC is a viable electrode to utilize in future energy-storage technologies [108].

## 5. Potential applications beyond supercapacitors

The multidimensional potential of TMCP-derived materials is demonstrated by their promising performance in further applications, including water splitting, batteries and electrocatalysis [20]. In water splitting applications, TMCP-derived materials demonstrate potential catalytic activity for oxygen evolution reaction (OER) and hydrogen evolution reaction (HER), with their variable composition and high surface area boosting catalytic efficiency [109,110]. A rationally built Ni–Co-based bimetallic MOF compounds was made by using glutaric acid as an organic ligand and low-temperature hydrothermal treatment that outperforms electrocatalytic and electrochemical charge storage activity in an alkaline medium. The NiCo-MOF outperformed other synthesized materials in the HER, with the lowest overpotential of 182  mV at 10 mA cm^−2^ of current density. It also demonstrated excellent kinetics, with a turnover frequency of 2.86  s^−1^, and greater stability after 1000 HER cycles in 1 M KOH. NiCo-MOF had a higher specific capacitance (978 F g^−1^) than Ni-MOF (706 F g^−1^) and Co-MOF (91 F g^−1^) at 1  A g^−1^ of current density. Theoretical studies revealed that Ni–Co sites provide excellent electronic coupling and a synergistic effect that improves the overall electronic characteristics of NiCo-MOF. As a result, its electrochemical performance in terms of water splitting and charge storage was improved. The study presents a novel method for creating ultrathin and flexible bimetallic MOF-based electrode materials that improve performance in electrocatalytic HER and supercapacitors [111]. CC@NC/NiCo-P is a freestanding 3D hierarchical nanostructure that is built for electrocatalytic hydrogen evolution and high-performance supercapacitors. The nitrogen-doped carbon (NC) layer, which is derived from polydopamine, acts as an interface coupling bridge to anchor electroactive NiCo-P nanowire arrays on flexible carbon cloth (CC) substrate. Because of the strong contact between the NiCo-P nanowires and the conductive carbon support, the resulting CC@NC/NiCo-P electrode has a capacity retention of 85.8% and an ultrahigh capacitance (2175.5 F g^−1^ at 1 A g^−1^). With an ultralong lifespan of 10 000 cycles, the constructed ASC attained an exceptional energy density of 28.47 Wh kg^−1^. Furthermore, the electrocatalytic activity of the CC@NC/NiCo-P electrode is favourable for the HER. The stability and electrochemical activity of CC@NC/NiCo-P are significantly enhanced by the strong interaction between the NC layer and metal species in TMPs, according to the research findings. This successful approach to creating novel electrode materials is anticipated to have promise for use in energy-related domains [112].

TMCPs can be used as building blocks for high-performance battery components, especially in lithium-ion and sodium-ion batteries, where their redox-active metal centres and porous architectures improve charge storage and ion diffusion [113]. With the help of atomic layer deposition, Luo *et al.* successfully grafted continuous mesoporous nanostructure Fe_3_O_4_ onto 3D graphene foams. The loose and porous structure of graphene alleviates the problem of shedding or pulverization of active materials caused by volume expansion. The composite material is used directly as the negative electrode of lithium-ion batteries, with a high reversible capacity and rapid discharge capability, with a capacity of up to 785 mAh g^−1^ at 1 C and stability over 500 charge and discharge cycles [20]. Hierarchical Na_3_V_2_(PO_4_)_3_/C microspheres perform exceptionally well in sodium-ion batteries regarding cyclic stability and high rate capabilities. Large surface area and many mesopores in this hierarchical layout allow for a short ion diffusion pathway and a substantial electrode–electrolyte interaction. The higher performance seen is the result of the combined influence of a large surface area, attractive structure integrity and the existence of a wide interstitial space [114]. Advanced water treatment technologies are aided by the exceptional adsorption capacities of some TMCP-derived materials for organic contaminants and heavy metal ions. Strong electrochemical reactions in TMCP-based materials make them appropriate for use in environmental sensors and biosensors that detect gases and biomolecules [67].

Since they can be designed into mechanically robust architectures and have high pseudocapacitance and structural flexibility, TMCP-based energy storage devices have great potential in the field of flexible electronics. The integration of TMCP-derived materials into super-foldable electrode systems by biomimetic techniques that mimic natural load-dispersing mechanisms has been the focus of recent study. For example, C/NiS nanofibre free-standing electrodes with intelligent stress dispersion and ultra-high mechanical resilience were made possible by the development of a two-level biomimetic structure modelled after spider silk and tendon microstructures. These electrodes retained over 90% of their capacitance after 50 000 folds and achieved an aerial capacitance exceeding 500 mF cm⁻² [115]. A conductive super-foldable material made from TMCPs was presented in another study. It combines carbon frameworks and transition metal sulfides in an elastic matrix that is hierarchically porous, leading to improved electrochemical performance and exceptional flexibility under continuous mechanical deformation [116]. The viability of incorporating TMCP-based materials into flexible, wearable energy devices was further demonstrated by the description of a biomimetic conductive nanostructure that supported remarkable electrochemical stability and structural integrity under extreme bending and folding conditions [117]. There are still issues to be resolved, including maximizing ion transport in compressed configurations, guaranteeing long-lasting electrochemical–mechanical coupling and scaling sustainable synthesis procedures, even if these investigations demonstrate the revolutionary potential of TMCPs in next-generation flexible electronics. However, with further advancements in flexible device integration and hierarchical material design, TMCP-derived systems have the potential to play a significant role in wearable and deformable energy storage technologies in the future.

## 6. Challenges and sustainable solutions

Although there has been significant advancement in the design and use of materials generated from TMCPs, quite a few issues still need to be resolved to guarantee their practical application in next-generation supercapacitors. The following subsections outline sustainable solutions to address the main constraints, such as those related to end-of-life management, environmental sustainability and scalable production.

### 6.1. Scalability of synthesis methods

The absence of scalable, affordable and ecologically friendly synthesis methods is one of the biggest obstacles to the commercial use of TMCPs. Even though traditional techniques like solvothermal, hydrothermal and pyrolysis approaches provide great control over shape and phase purity, they are difficult to modify for mass production and frequently call for severe conditions (such as high temperatures, lengthy reaction times and hazardous solvents). Developing scalable and cost-effective synthesis methods remains a significant challenge. Researchers are beginning to develop scalable and environmentally friendly solutions to these obstacles, such as low-temperature pathways and water-based systems, which drastically cut down on energy use and environmental effect [33,55,118]. One example is the effective production of MnO₂ nanoparticles (NPs) at room temperature using plant extracts. After adding a natural reducing agent made from plant extract to a solution of KMnO₄, the colour changed from purple to black, signifying full reduction in just 1 h. α-MnO₂ NPs were obtained by washing, drying at 90°C, and annealing at 300°C. These substances demonstrated encouraging uses in lithium batteries, supercapacitors and photocatalysis [33].

### 6.2. Environmental concerns and sustainability

Adding to the requirement for scalable techniques, the environmental impact of mining, processing and discarding the transition metals utilized in TMCPs is another major obstacle. Particularly when non-renewable or toxic precursors are used, these operations have the potential to produce hazardous waste and deplete natural resources [118,119]. Eco-friendly synthesis techniques are being investigated to mitigate these worries. For instance, Hashem *et al.* used lemon juice and lemon peel as reducing agents to demonstrate the environmentally friendly synthesis of MnO₂ NPs, producing J-MnO₂ and P-MnO₂, respectively. These substances were tested for usage as cathode materials in lithium-ion batteries and were synthesized without the production of harmful byproducts. These tactics highlight the benefits of substituting traditional hazardous reagents with reactants produced from biomass, hence increasing the sustainability of TMCP synthesis [33,118,119].

### 6.3. Green chemistry and recycling strategy

To enhance sustainability, the complete life cycle of TMCP-based materials must be examined, rather than only synthesis. This includes designing materials for end-of-life management and incorporating green chemistry ideas into processing and recycling [120]. One viable technique is to use renewable waste sources as both carbon templates and reducers. Abuzeid *et al.* produced MnO₂ nanoparticles from orange peel extract, recycling agricultural waste and making excellent supercapacitor electrodes [33]. Furthermore, developing TMCPs with disassembly, recyclability and low component incompatibility can help these materials achieve their circular economy promise. Hydrothermal technologies employing non-toxic, recyclable solvents and structure-preserving recovery processes should be prioritized in future research efforts. Improving the scalability, environmental sustainability and recyclability of TMCP-derived materials is critical for their transition from laboratory-scale innovation to real-world applications. A comprehensive approach incorporating green synthesis, lifespan analysis and recycling techniques will ensure that these promising materials are consistent with global sustainability objectives [11,120–124].

## 7. Future perspectives

As investigations on TMCPs advance, there are still a few scientific and technological directions that could potentially be pursued. Transforming TMCP-based supercapacitors from laboratory-scale breakthroughs to scalable, dependable and commercially viable devices will require addressing these issues. This section highlights recent developments and foresees the main obstacles that scientists handling this intricate material system will probably encounter.

### 7.1. Predictive modelling, machine learning and high accuracy screening

The lengthy development cycles caused by the experimental process for material selection and synthesis optimization are one of the persisting problems in TMCP research. This problem is currently being addressed by machine learning (ML) and artificial intelligence (AI), which forecast material stability, ideal synthesis pathways and structure–property connections. For certain performance goals, ML models trained on databases of known TMCPs and their performance metrics can recommend new morphologies and compositions. High accuracy experimental and computational screening (e.g. DFT, molecular dynamics) in conjunction with ML can also facilitate the quick identification of TMCPs with optimal conductivity, redox activity and structural stability. Scientists must, however, overcome the absence of widely recognized material identifiers for coordination polymer systems, variable reporting standards and a lack of data for these methods to be successful. Researchers will have to build intelligent TMCP design frameworks in the future by curating extensive, high-quality databases, combining ML with *in situ* diagnostics and working across disciplines [121,125,126].

### 7.2. Emerging materials and novel chemistries for next-generation TMCPs

Exciting prospects are presented by an increasing number of novel materials and hybrid systems that go beyond the current TMCP formulations. Among them are coordination structures with a bioinspired design that are sustainable and biocompatible and based on nucleotides, peptides or amino acids. MXenes and 2D heterostructures offer better mechanical robustness and electrical conductivity. For ion diffusion and electron transport, covalent organic frameworks and metal–organic cages provide highly adjustable pore shapes [67,103,127]. Interfacial stability, heterogeneity and synthesis repeatability are issues that arise when incorporating these novel materials into TMCP-based designs. Furthermore, novel redox chemistries that use multiple electrons or linked cation/anion storage may boost specific capacitance, but they also carry the risk of parasitic side reactions or structural collapse. Future studies must concentrate on improving component compatibility, promising long-term structural integrity and figuring out atomic and molecular degradation mechanisms [67,127].

### 7.3. Real-world application: device engineering, scale-up and stability

Even though laboratory-scale TMCP materials frequently exhibit remarkable electrochemical qualities, they need to be designed into full-cell devices that adhere to industrial standards. Important challenges for scientists include retaining the same shape and electrochemical performance as laboratory-scale samples while synthesizing TMCPs at scale is known as reproducibility across batches. Changing the electrode format from powder to flexible, 3D-printed or binder-free should be done without sacrificing conductivity. Under high-voltage or prolonged cycle conditions, TMCPs must be stable in a variety of aqueous, organic or gel electrolytes. It will also be necessary to use life-cycle assessment frameworks, circular material recovery and green synthesis to address the environmental effects, toxicity and precursor costs of large-scale TMCP production [128–130].

## 8. Conclusion

The rapid development of TMCP materials is transforming the landscape for next-generation supercapacitor electrodes. TMCPs provide unparalleled control over composition, shape and electrochemical behaviour by combining the redox tunability of transition metals with the modularity of coordination frameworks. Significant achievements have been made in hierarchical nanostructures, hybrid composites and post-synthetic alterations (e.g. sulfidation, phosphorization, carbonization), all of which aim to improve conductivity, stability and charge storage capacity. These methodologies have enabled some TMCP-based devices to attain specific capacitances surpassing 1000 F g⁻¹, combined with good cycle and rate performance. Despite this, there are still limitations with long-term stability, electrolyte compatibility, environmental sustainability and scale-up. A paradigm change from performance-focused laboratory research to comprehensive system design and life cycle evaluation is required for TMCP-based supercapacitors to become commercially feasible. Unlocking the full potential of TMCPs will need an intersection of green chemistry, machine learning and multifunctional hybrid materials. Multidisciplinary cooperation between materials science, electrochemistry, data science and environmental engineering is the way forward to realize the potential of materials developed from TMCPs for a high-performance, sustainable energy storage future.

## Data Availability

This article has no additional data.
